# Fiber-Reinforced One-Part Geopolymer Mortars Incorporating Red Mud, Ceramic Powder, and MgO: Performance Under Different Curing Regimes and Curing-Based Environmental Assessment

**DOI:** 10.3390/polym18162023

**Published:** 2026-08-20

**Authors:** Mohammed Dakhel Al Bdairi, Orhan Canpolat, Mucteba Uysal, Ömer Can Özen, Ömer Faruk Kuranlı, Aygül Zara Kebir

**Affiliations:** Faculty of Civil Engineering, Department of Civil Engineering, Yıldız Technical University, 34220 Istanbul, Türkiye; canpolat@yildiz.edu.tr (O.C.); mucteba@yildiz.edu.tr (M.U.); omer.ozen@yildiz.edu.tr (Ö.C.Ö.); faruk.kuranli@std.yildiz.edu.tr (Ö.F.K.); zara.kebir@yildiz.edu.tr (A.Z.K.)

**Keywords:** one-part geopolymer, micro-fibers, life cycle assessment, industrial by-products, MgO, durability, heat curing

## Abstract

**Featured Application:**

The developed one-part geopolymer mortar may be considered for precast or wear-exposed mortar products that can benefit from one-part alkali activation and fiber reinforcement. Its use in repair mortars, overlays, paving, or other service applications remains conditional on further testing of storage stability, open time, bond strength, shrinkage, cracking, freeze-thaw resistance, and substrate compatibility.

**Abstract:**

One-part geopolymer mortars provide an alternative to cementitious materials by using dry activators and industrial by-products. This study evaluated a multi-precursor matrix containing slag, fly ash, ceramic powder, red mud, and 5% MgO, reinforced with polyvinyl alcohol (PVA), basalt, or micro-steel fibers at 0.4% and 0.8% by volume. Specimens were cured at 20 ± 2 °C or at 80 °C for 24 h and assessed for flowability, mechanical properties, ultrasonic pulse velocity (UPV), Böhme abrasion, 24 h water absorption, and sorptivity. XRD, FTIR, and SEM/EDS were used only to compare by heat-cured mixtures. The results showed that at 28-day, heat curing increased compressive strength by 39.5–71.8%, flexural strength by 29.2–134.4%, and UPV by 20.5–36.4%, while reducing sorptivity by 12.2–35.7% relative to ambient curing. PVA reduced flowability likely because of its hydrophilic surface and high surface area. For 0.8PVA, the flow diameter was 23.6% below the reference, whereas the 28-day heat-cured flexural strength reached 7.5 MPa, with the lowest abrasion thickness loss of 0.63 mm. Micro-steel mixtures maintained compressive strength comparable to the reference, reaching 72–73 MPa at 28-day. Heat curing reduced water absorption in PVA and basalt mixtures, whereas the reference and micro-steel mixtures showed insignificant change. Microstructural analyses suggested the formation of a more compact and reacted aluminosilicate matrix under heat-cured conditions. A screening life-cycle assessment, limited to the non-fiber-reinforced reference matrix, showed that heat-curing increased global warming potential by 7.6%, while the two solid activators contributed approximately 45% of the ambient-cured reference GWP. The findings indicate that heat curing significantly enhances the performance of one-part geopolymers, while fiber selection provides additional mechanical and durability improvements.

## 1. Introduction

Geopolymer and alkali-activated binders have been widely investigated as lower-clinker alternatives to Portland cement because they can utilize industrial by-products and reduce the environmental burden associated with conventional cement manufacture [1,2,3]. Among the available approaches, one-part geopolymers are particularly attractive for practical implementation because the alkaline components are supplied in dry form and the material can be prepared by addition of water only, which improves handling safety and simplifies transportation, storage, and field application [4,5].

The performance of one-part geopolymer mortars depends on the combined effects of precursor chemistry, solid activator composition, curing regime, and reinforcement strategy. Elevated-temperature curing is commonly used to accelerate dissolution of reactive phases and promote earlier formation of a dense binding matrix [2,6]. Fiber incorporation, on the other hand, may improve crack control, tensile response, and post-cracking behavior, although its effectiveness strongly depends on fiber geometry, stiffness, surface characteristics, dosage, and compatibility with the geopolymer matrix [7,8]. Therefore, the response of fiber-reinforced one-part geopolymer mortars cannot be interpreted only from the individual contribution of the matrix or the fibers, but should be evaluated considering their interaction under different curing conditions.

In parallel with these developments, increasing attention has been directed toward the use of industrial residues such as red mud and ceramic powder in alkali-activated systems. These materials are particularly relevant not only because of their potential contribution to resource efficiency and waste valorization, but also because their aluminosilicate-rich composition may participate in or support geopolymerization reactions [9,10]. In slag-based systems, the incorporation of MgO may further influence reaction development and microstructural refinement, particularly through secondary reaction products reported in previous studies [11,12,13]. Existing studies have largely addressed fiber-reinforced alkali-activated systems using conventional two-part liquid activators or individual precursor combinations, with limited attention to multi-precursor one-part formulations incorporating red mud and ceramic powder simultaneously under contrasting curing regimes [7,8,9,10]. However, in multi-precursor systems containing slag, fly ash, ceramic powder, red mud, and MgO, the matrix is chemically complex, and the influence of external variables such as curing regime and fiber type becomes more difficult to predict.

Recent advances in one-part geopolymer technology have demonstrated that multi-component precursor systems combining ground granulated blast-furnace slag (GGBFS), fly ash, and supplementary industrial residues can achieve compressive strengths ranging from 30 to 80 MPa depending on activator dosage and curing conditions [14,15]. Systems activated with solid sodium metasilicate and sodium carbonate have been shown to develop adequate early strength and workability without the handling hazards associated with liquid alkaline solutions, while maintaining a lower carbon footprint relative to conventional two-part formulations [16,17,18]. The incorporation of reactive MgO as a minor additive has been reported to refine the binding matrix and promote formation of secondary hydrotalcite phases in slag-rich systems, contributing to improved microstructural density [13]. Similarly, red mud and ceramic powder have been successfully valorized as partial precursor replacements in alkali-activated mortars, with their high alumina and silica contents supporting geopolymerization reactions and enhancing durability performance [10,11,19,20].

Previous studies on fiber-reinforced one-part geopolymers have generally focused on single-precursor or binary binder systems, individual fiber types, or a single curing condition, with limited direct comparison of PVA, basalt, and micro-steel fibers within the same multi-precursor chemistry [4,14]. These fibers differ substantially in elastic modulus, surface characteristics, and fiber–matrix interaction, and their relative effectiveness under ambient and heat-curing conditions remains insufficiently understood [7,8,21]. Therefore, the novelty of the present study does not arise merely from combining previously investigated constituents. Its principal contribution is the controlled comparison of fiber type and dosage under ambient and heat-curing conditions within a fixed one-part matrix containing slag, fly ash, ceramic powder, red mud, and MgO. By keeping the binder and activator composition constant, the study identifies property-specific trade-offs among flowability, mechanical performance, abrasion resistance, water absorption, and sorptivity rather than proposing a universal ranking of fiber types. Furthermore, few studies have extended the evaluation of one-part geopolymer mortars to include abrasion resistance and capillary water absorption alongside standard mechanical testing, leaving gaps in the durability characterization required for practical deployment in repair and precast applications [15,22]. A secondary contribution is the screening comparison of the performance benefits and additional environmental burden associated with heat curing for the non-fiber-reinforced reference matrix.

Accordingly, the study tested the hypothesis that heat curing would primarily affect matrix development by strength and UPV, whereas fiber type and dosage would modify mechanical response, surface wear resistance, and water-transport behavior according to fiber geometry and surface characteristics. To test this hypothesis, this study evaluated the effects of fiber type and curing regime on a one-part geopolymer mortar produced from multiple industrial aluminosilicate precursors. Therefore, the experimental program covered flowability, flexural and compressive strength, splitting tensile strength, UPV, and durability-related indicators comprising Böhme abrasion, 24 h water absorption, and sorptivity. The test results were supported by XRD, FTIR, and SEM/EDS analyses, which were used only to compare heat-cured mixtures. Particular attention is given to identifying the fiber systems that provide the most favorable balance between strength development, durability-related behavior and practical applicability.

## 2. Materials and Methods

### 2.1. Raw Materials

The geopolymer mortar was produced using multiple industrial aluminosilicate precursors, including ground granulated blast furnace slag (GGBFS), fly ash, ceramic powder, and red mud, in line with previous studies reporting the effective utilization of industrial by-products in alkali-activated and geopolymer systems to reduce environmental impact and clinker dependency [1,2,3,4,5,6,7,8,9,10,11,12,13,16,20,23].

The red mud was obtained as a by-product of alumina production from ETI Alüminyum A.Ş. (Konya, Türkiye), as similarly reported in geopolymer studies incorporating red mud as a supplementary precursor or filler [9,10]. The red mud was received in bulk form, oven-dried to remove residual moisture, and mechanically ground to fine powder (<90 μm) at Polsan Paint Factory (Kocaeli, Türkiye), following procedures commonly adopted to enhance reactivity and homogeneity of red mud in geopolymer matrices [9,10].

Reactive magnesium oxide (MgO) was incorporated as a supplementary mineral additive to regulate reaction kinetics and improve microstructural refinement, as documented in alkali-activated and geopolymer binders containing MgO [11,12,13].

A dry alkaline activator system was employed to enable one-part geopolymerization, allowing the mortar to be prepared by the addition of water only, which has been shown to significantly enhance handling safety and practical applicability compared to conventional liquid activators [4,5,6]. The solid activator consisted of sodium metasilicate pentahydrate (Na_2_SiO_3_·5H_2_O) and sodium carbonate (Na_2_CO_3_), a combination widely reported for one-part geopolymer systems [4,5]. Sodium metasilicate pentahydrate was mechanically ground prior to use to improve dissolution rate and reactivity, as recommended in previous one-part geopolymer studies [5,23]. The adoption of solid alkaline activators was intentionally selected to reduce health and environmental risks associated with alkaline solutions and to improve the sustainability and field applicability of the geopolymer system [4,5,6].

Standard sand conforming to EN 196-1 was used as fine aggregate.

To investigate the influence of fiber reinforcement on the physical and mechanical performance of the geopolymer mortar, polyvinyl alcohol (PVA), basalt, and micro-steel fibers were incorporated at volume fractions of 0.4% and 0.8%, consistent with previous studies on fiber-reinforced geopolymer mortars [7,8]. Micro-steel fibers were obtained from Fiberego (Jinan, China), whereas PVA and basalt fibers were obtained from local construction material importers. The detailed physical and mechanical properties of the fibers are summarized in Table 1.

**Figure 1:**
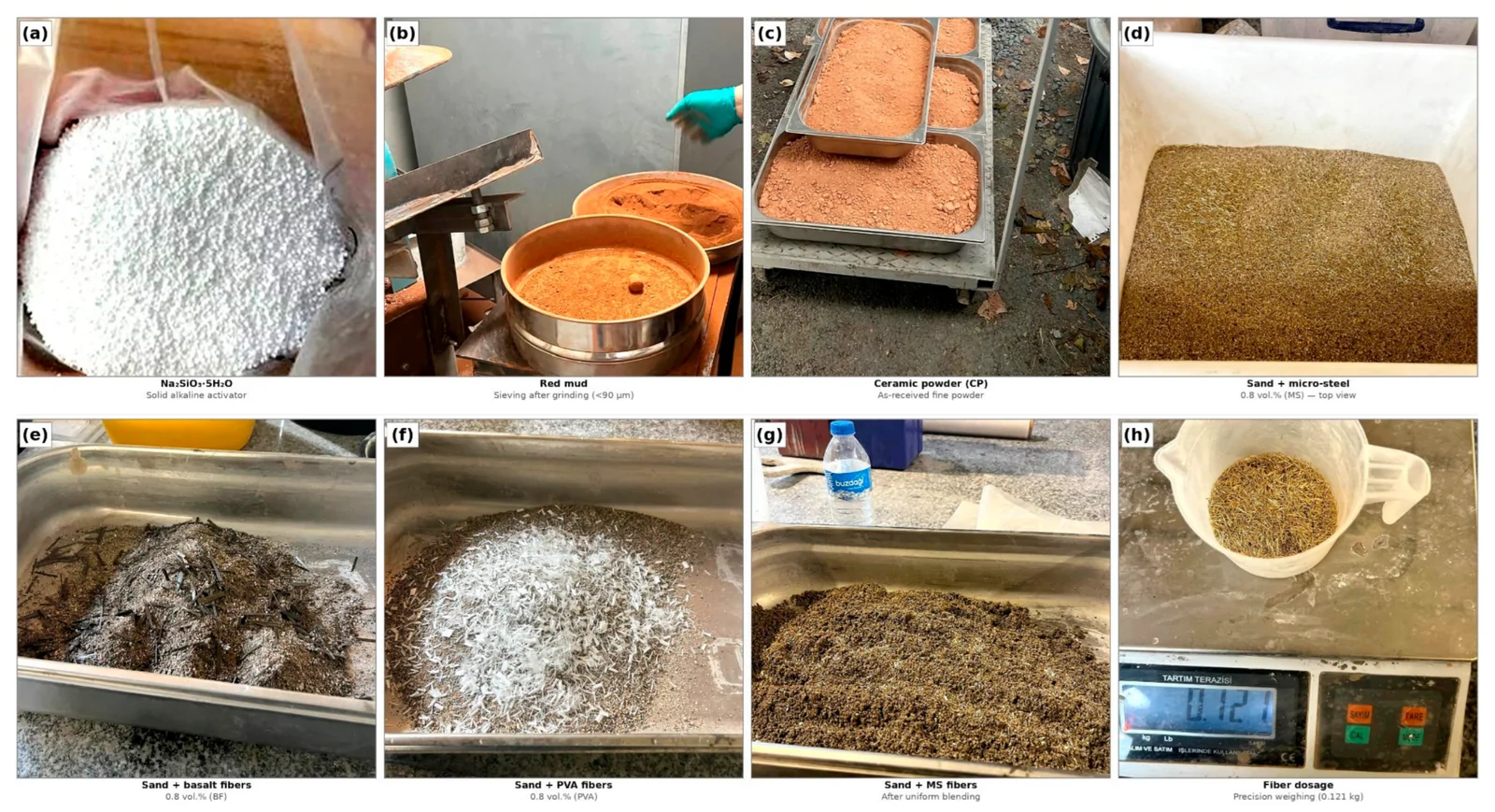
Representative raw materials and fiber-sand pre-blending: (**a**) sodium metasilicate pentahydrate (Na_2_SiO_3_·5H_2_O); (**b**) ground and sieved red mud (<90 μm); (**c**) ceramic powder (CP); (**d**–**h**) pre-blending and dosage of micro-steel, basalt and PVA fibers.

The chemical compositions of the raw precursor materials, as determined by X-ray fluorescence (XRF) analysis at the Yıldız Technical University Central Laboratory (İstanbul, Türkiye), are summarized in Table 2. The XRF results of MgO, also presented in Table 2, were obtained from an unpublished technical datasheet provided directly by representatives of Omsan Maden (İzmir, Türkiye) through personal communication. The reference mix formulation was selected based on preliminary trials aimed at achieving adequate fresh workability and sufficient early mechanical performance without segregation or excessive water demand. Since a single multi-precursor matrix was adopted, the effects reported in this study should be interpreted within the compositional framework of the selected slag-fly ash-ceramic powder-red mud-MgO system. Representative photographs of the raw materials and the fiber-sand pre-blending procedure are presented in Figure 1.

### 2.2. Mix Proportions and Mixing Procedure

The mix design of the geopolymer mortars is summarized in Table 3. All mixtures were prepared with a constant water-to-binder ratio of 0.32 and a sand-to-binder ratio of 2:1. The mixtures were designated according to the fiber type incorporated in their composition as “PVA”, “BF”, and “MS”. Fiber reinforcement was incorporated at volume fractions of 0.4% and 0.8%.

While the baseline formulation was initially guided by preliminary trial mixtures, the specific binder proportions were scientifically formulated to optimize reaction kinetics, workability, and microstructural integrity. The binder proportions were scientifically formulated to optimize reaction kinetics, workability, and microstructural integrity. GGBFS (50%) was utilized as the primary calcium source to drive early C–A–S–H gel formation and matrix densification [25,26]; however, its dosage was restricted to 50% to prevent the excessive drying shrinkage and flash-setting behavior typically associated with pure slag systems [27]. To counterbalance this rapid setting, fly ash (25%) was incorporated. The spherical morphology of fly ash provides a ‘ball-bearing’ effect that enhances fresh workability despite the low water-to-binder ratio (0.32) [28], while its reactive aluminosilicates support later-age N–A–S–H gel development [29]. Furthermore, ceramic powder and red mud were restricted to 10% each to reduce the environmental footprint without causing a severe ‘dilution effect’. Replacing reactive precursors with these less reactive by-products beyond 10–15% is known to disrupt gel continuity, increase porosity, and significantly degrade mechanical strength [25]. 5% MgO was included to mitigate drying shrinkage and refine the macro-pore structure via the formation of expansive secondary phases, such as hydrotalcite. This 5% dosage serves as an optimal threshold, as higher MgO contents would excessively dilute the reactive GGBFS fraction, thereby negatively affecting the matrix porosity and water absorption properties [30]. Similarly, a hybrid solid activator comprising 12% sodium metasilicate and 4% sodium carbonate was selected. While the metasilicate ensures adequate precursor dissolution and polycondensation, relying solely on it significantly increases the carbon footprint and induces rapid setting. The incorporation of 4% sodium carbonate mitigates these issues by lowering the overall global warming potential and extending the open time for adequate workability [31,32]. Finally, a water-to-binder ratio of 0.32 was adopted. This ratio provides sufficient moisture for the complete dissolution of solid activators without inducing excessive capillary porosity, aligning with literature that identifies 0.28–0.32 as the optimum range for achieving a dense gel network and ideal rheology in silicate-activated slag systems [25,33].

The geopolymer mortar was prepared using a standard laboratory mortar mixer with two operating speeds (approximately 140 rpm for slow mixing and 285 rpm for high-speed mixing). The mixing procedure was designed to ensure uniform dispersion of the dry constituents and adequate homogenization of the fresh geopolymer mortar.

Initially, the dry precursor materials and powdered alkaline activators were introduced into the mixer and blended for approximately 2 min at low speed (~140 rpm) to achieve uniform distribution of the solid components. Subsequently, the mixing water was gradually added over approximately 30 s while the mixer was operating.

After the initial wetting of the binder phase, standard sand with fibers was progressively incorporated into the mixture at a binder-to-sand ratio of 1:2, while the mixer continued to operate for approximately 1 min. Following the addition of all constituents, the fresh mortar was mixed for 3 min to obtain a homogeneous mixture.

### 2.3. Specimen Preparation and Curing

Prismatic specimens (40 × 40 × 160 mm) were prepared for flexural and compressive strength testing, while cylindrical specimens (Ø100 × 200 mm) were prepared for splitting tensile strength evaluation. Cubic specimens (71 × 71 × 71 mm) were cast for abrasion resistance and water absorption testing.

**Figure 2:**
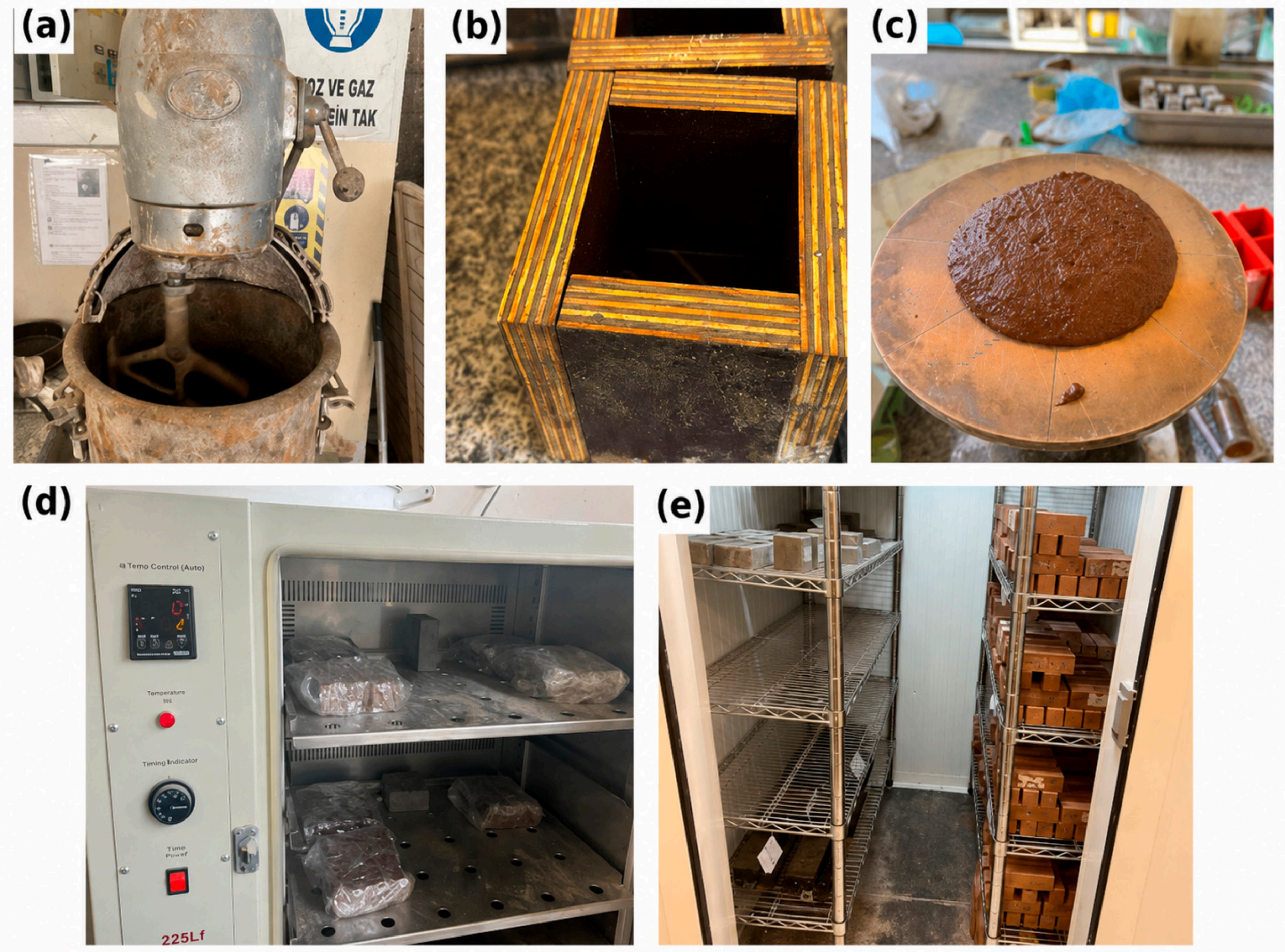
Specimen fabrication and curing sequence: (**a**) mechanical mixer; (**b**) wooden cubic molds (71 × 71 × 71 mm); (**c**) flow table test for workability assessment; (**d**) thermal curing at 80 °C for 24 h; (**e**) specimens stored in humidity-controlled curing chamber.

After casting, all specimens were stored at room temperature for 24 h under sealed conditions to prevent moisture evaporation. Following demoulding, specimens were divided into two groups: one group was subjected to ambient curing (20 ± 2 °C) until the designated testing age, while the other group was exposed to thermal curing at 80 °C for 24 h under sealed heat-resistant bags to minimize moisture loss during curing. The overall specimen fabrication and curing procedure, encompassing mechanical mixing, casting, demoulding, workability assessment, thermal treatment and subsequent humidity-controlled storage, is presented in Figure 2.

### 2.4. Flowability Test

The flowability of the fresh geopolymer mortar was evaluated using a flow table test in accordance with ASTM C1437, a standard method widely applied for assessing the workability of cementitious and geopolymer mortars [34]. The average spread diameter after 25 table drops within 15 s was recorded.

### 2.5. Mechanical Testing

**Figure 3:**
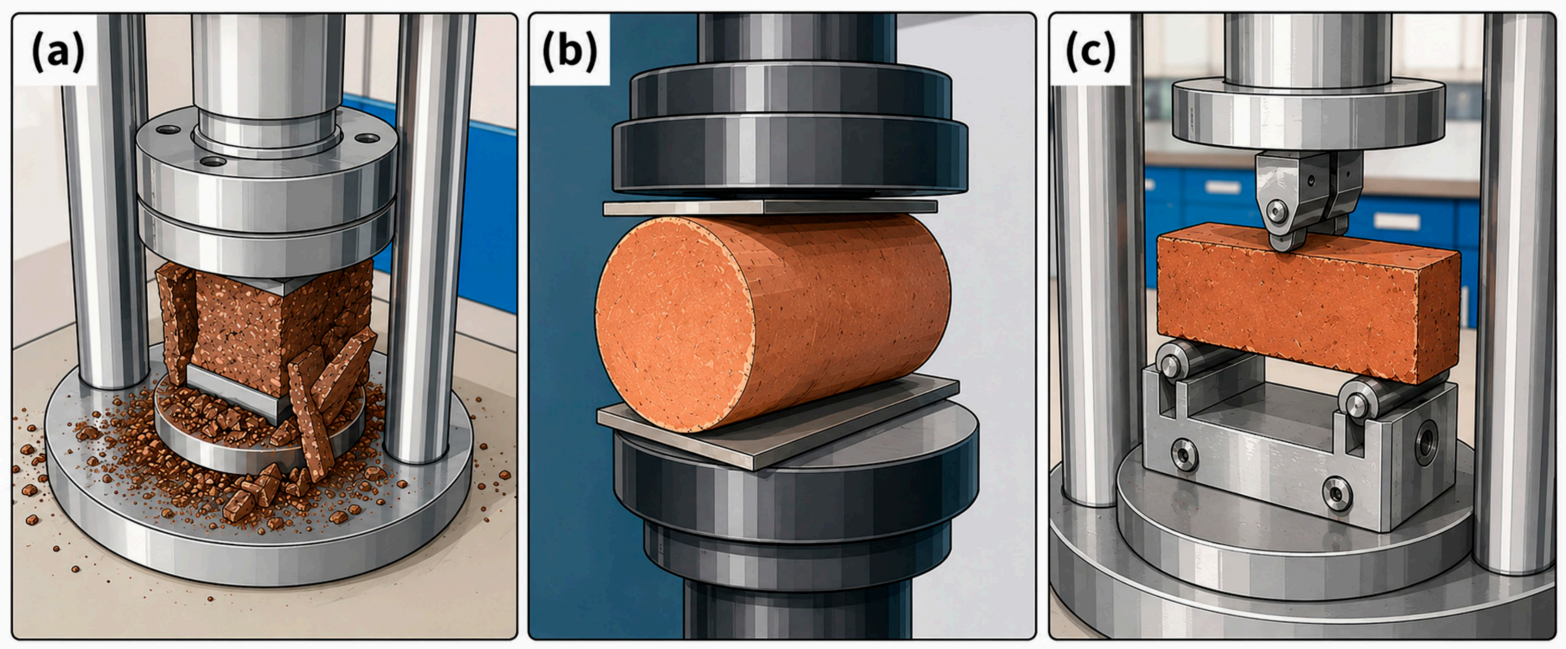
Laboratory testing apparatus: (**a**) compressive strength testing machine with failed specimen; (**b**) splitting tensile (Brazilian) test setup; (**c**) flexural (three-point bending) strength testing machine.

The laboratory testing apparatus used for the mechanical characterization of the specimens is illustrated in Figure 3, including the compressive strength test setup (Figure 3a), splitting tensile test setup (Figure 3b), and flexural strength test setup (Figure 3c).

#### 2.5.1. Compressive and Flexural Strength

Compressive and flexural strength tests were conducted in accordance with EN 196-1 using a calibrated universal testing machine. The reported values represent the average of three specimens for each mixture.

#### 2.5.2. Splitting Tensile Strength

The splitting tensile strength was determined using the Brazilian test method in accordance with EN 12390-6, which is widely adopted for evaluating the tensile performance of concrete and geopolymer materials [35,36]. Cylindrical specimens (Ø100 × 200 mm) were positioned horizontally between loading platens. The load was applied at a constant rate of 1.50 kN/s until failure, and the splitting tensile strength was calculated using:

*f ct,sp* = 2*P*/(*πLD*)

where f ct,sp is the splitting tensile strength (MPa), P is the maximum applied load (N), L is the specimen length (mm), and D is the specimen diameter (mm).

### 2.6. Ultrasonic Pulse Velocity (UPV) Test

Ultrasonic pulse velocity measurements were performed in accordance with ASTM C597 to assess internal quality and homogeneity of the geopolymer mortar, as commonly applied in alkali-activated materials [37]. Measurements were conducted on 40 × 40 × 160 mm prismatic specimens before flexural testing, using the direct transmission method along the longitudinal axis of the specimens. Three measurements were taken for each mixture and curing regime, and the mean value was reported.

### 2.7. Water Absorption and Capillary Water Absorption

Capillary water absorption was evaluated following ASTM C1585, with a modified specimen geometry adapted for geopolymer mortars [38,39]. Cubic specimens (7.1 × 7.1 × 7.1 cm) were oven-dried at 105 °C for 24 h, cooled to room temperature, and sealed on the top surface and four lateral faces using adhesive tape, leaving one face exposed to water. Mass gain was recorded at predefined time intervals up to 24 h, and the sorptivity coefficient was determined from the slope of the initial linear portion of the cumulative absorption versus square root of time curve, as recommended in previous studies [38,39].

### 2.8. Abrasion Resistance (Böhme Test)

Abrasion resistance was assessed using a Böhme abrasion tester in accordance with EN 13892-3, a standard method frequently used to evaluate surface wear resistance of mortar-based materials [40,41,42]. Cubic specimens (7.1 × 7.1 × 7.1 cm) were centrally loaded with 294 ± 3 N and subjected to 16 abrasion cycles, each consisting of 22 revolutions. Artificial corundum abrasive powder (20 g) was applied to the rotating steel disc operating at 30 rpm. After each cycle, the abrasive material was renewed, and the specimen was rotated by 90° to ensure uniform wear. Abrasion loss was determined by measuring thickness reduction at six predefined points, and the average value was reported. The Böhme abrasion testing apparatus used to evaluate the abrasion resistance of the specimens is shown in Figure 3b.

### 2.9. XRD, FTIR, and SEM/EDS Characterization

To further investigate the phase composition, bond structure, and microstructural features of the developed one-part geopolymer mortars, selected specimens were characterized using X-ray diffraction (XRD), Fourier transform infrared spectroscopy (FTIR), and scanning electron microscopy coupled with energy-dispersive spectroscopy (SEM/EDS).

XRD analysis was performed on heat-cured specimens M1 (Ref), M2 (0.8PVA), M3 (0.8BF), and M4 (0.8MS) using a PANalytical Empyrean diffractometer with Cu-Kα radiation (λ = 1.5406 Å), operated at 45 kV and 40 mA, over a 2θ range of 5–70°, with a step size of 0.013°. XRD was employed to identify the main crystalline features and evaluate the persistence of residual crystalline phases within the geopolymer matrix, as commonly reported for alkali-activated and geopolymer materials [43,44].

FTIR spectra were recorded for the same heat-cured specimens (M1–M4) to identify the main functional groups and vibration bands associated with geopolymeric gel formation and carbonate-containing phases. FTIR is widely used in geopolymer studies to monitor the evolution of the Si–O–T band (T = Si or Al), which reflects the structural reorganization of the aluminosilicate network during geopolymerization [44,45].

SEM/EDS observations were conducted on selected representative specimens to assess matrix compactness, the presence of unreacted particles, pore distribution, and local elemental composition. SEM imaging was used to qualitatively compare the morphology of the hardened matrix, while EDS served as a complementary tool to verify the presence of major elements associated with the slag–fly ash–ceramic powder–red mud-based geopolymer system [43,44]. Because the microstructural analyses were conducted on selected heat-cured specimens, these results were used mainly to support the comparison among fiber types under the selected curing condition and were not intended to isolate the full microstructural effect of the curing regime.

It should be noted that the microstructural analyses were conducted only on the heat-cured M1–M4 specimens. Therefore, these analyses were intended to compare the effects of fiber type under a common curing condition and do not provide direct microstructural evidence of the differences between heat and ambient curing.

### 2.10. Life Cycle Assessment Methodology

**Figure 4:**
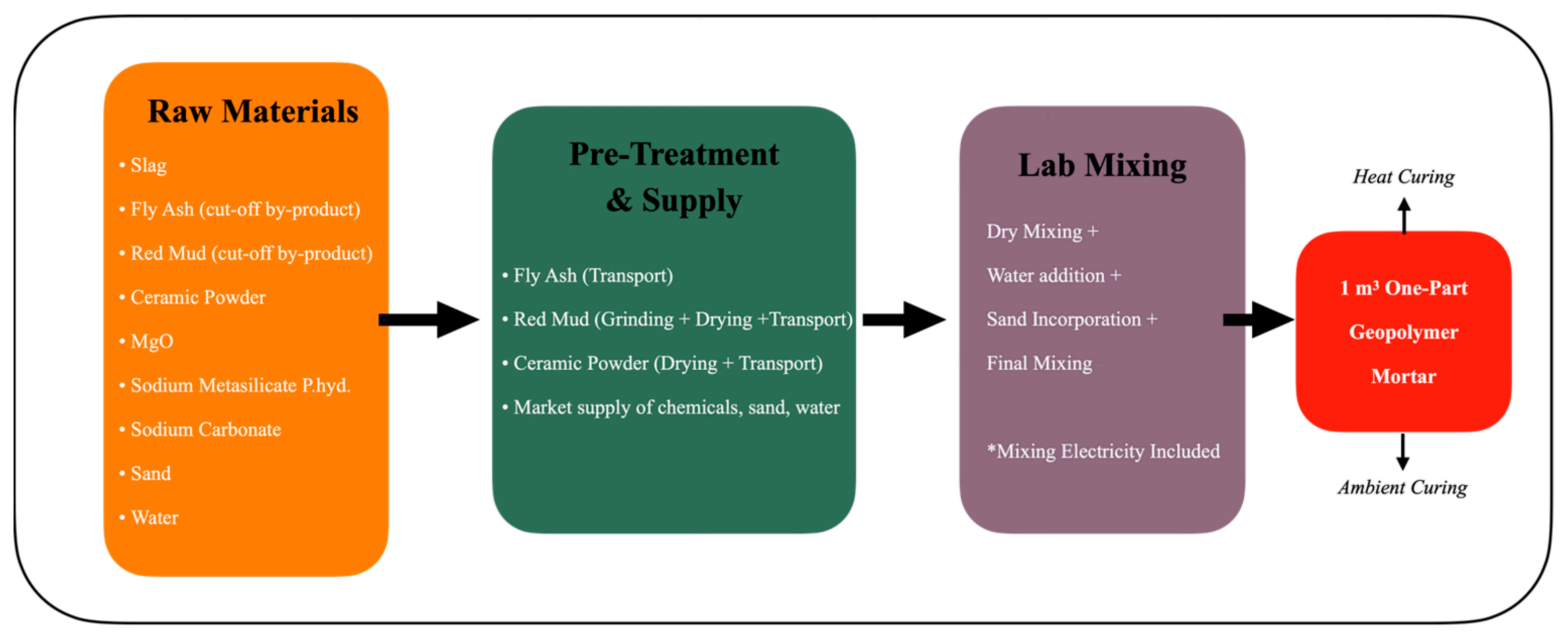
Schematic representation of the cradle-to-gate LCA system boundary for the reference one-part geopolymer mortar, including raw materials, pre-treatment and supply processes, laboratory mixing and production of 1 m^3^ fresh mortar. *: Mixing electricity included.

A cradle-to-gate screening life cycle assessment was conducted using SimaPro PhD 10.3 licensed software and the Ecoinvent 3.11 database to evaluate the environmental profile of the reference one-part geopolymer mortar. System boundary and main inventory flows considered in the LCA are schematically presented in Figure 4. The functional unit was defined as 1 m^3^ of fresh mortar. The system boundary included raw material supply, production of solid alkaline activators, sand and water supply, pre-treatment of waste-derived materials, local transportation assumptions for selected by-products and electricity consumption for laboratory mixing. For the heat-cured scenario, electricity consumption associated with oven curing at 80 °C for 24 h was additionally included. Moulds, laboratory equipment, mechanical testing, packaging, use phase and end-of-life stages were excluded from the analysis.

Environmental impacts were calculated using the CML-IA baseline method in SimaPro. The selected impact categories were abiotic depletion, abiotic depletion of fossil fuels, global warming potential (GWP100a), ozone layer depletion, human toxicity, freshwater aquatic ecotoxicity, marine aquatic ecotoxicity, terrestrial ecotoxicity, photochemical oxidation, acidification and eutrophication. Thus, the assessment was not limited to global warming potential but also covered resource depletion, toxicity-related impacts, atmospheric emissions and eutrophication effects. For the ambient-cured reference mixture, the contribution of each material and process to each impact category was quantified and reported in tabular form. For the heat-cured reference mixture, the total characterized results for each impact category were compared with the corresponding ambient-cured values. The percentage variation between the two scenarios was then calculated and presented graphically to evaluate the additional environmental burden associated with heat curing. In addition to tabular contribution results, a SimaPro process network was generated for the ambient-cured reference mixture to visualize the main inventory flows and dominant contributors within the product system. This network diagram was used as a supplementary interpretation tool to trace the contribution of raw materials, pre-treatment processes, transport and electricity inputs to the overall characterized result.

To clarify the scope of the environmental interpretation, Table 4 summarizes the impact categories evaluated using the CML-IA baseline method and the approach adopted to interpret the ambient and heat-curing scenarios.

The LCA was performed for the reference mixture under ambient and heat-curing conditions. This approach was adopted to establish a clear environmental baseline for the one-part geopolymer matrix and to evaluate the additional environmental burden associated with thermal curing. Since the reference formulation contains the same binder system, activator composition and waste-derived precursors that form the basis of the fiber-reinforced mixtures, it provides a representative indication of the main environmental hotspots of the material system. Fiber-reinforced mixtures were not included in the baseline LCA because representative datasets for the specific PVA, basalt and micro-steel fibers used in this study were not available in Ecoinvent 3.11. Their inclusion would therefore require proxy assumptions with high uncertainty, which could reduce the robustness of the comparative interpretation.

Fly ash, red mud and ceramic powder were modelled under a cut-off allocation approach. Therefore, no upstream environmental burdens from their original production systems were assigned to these materials. Instead, only the processes required to make them suitable for mortar production were considered. Fly ash was modelled with drying electricity and 30 km transportation by light commercial vehicle. Red mud was modelled with drying and grinding electricity, reflecting the fact that it was received in bulk form, oven-dried and mechanically ground to a fine powder before use. Ceramic powder was modelled as a waste-derived powder, with drying electricity and 30 km transportation included, while crushing and grinding were excluded because the material was received in powder form. The 30 km transport distance was adopted as a local transport assumption based on approximate distances within Istanbul between material suppliers, processing locations and the laboratory.

The remaining raw materials, including GGBFS, MgO, sodium metasilicate pentahydrate, sodium carbonate, standard sand and water, were modelled using corresponding or closest available RoW datasets from Ecoinvent 3.11, since Türkiye-specific datasets were not available. Sodium carbonate was modelled using the dataset for soda ash, light, while standard sand was represented using the sand or silica sand dataset as the closest available proxy. Mixing water was modelled using the tap water dataset. This modelling strategy was adopted to maintain consistency across the inventory and to avoid unsupported country-specific substitutions where no representative local datasets were available. To improve transparency and reproducibility, the main assumptions adopted for process modelling, transportation and dataset selection are summarized in Table 5.

## 3. Results

### 3.1. Flowability of Fresh Geopolymer Mortars

**Figure 5:**
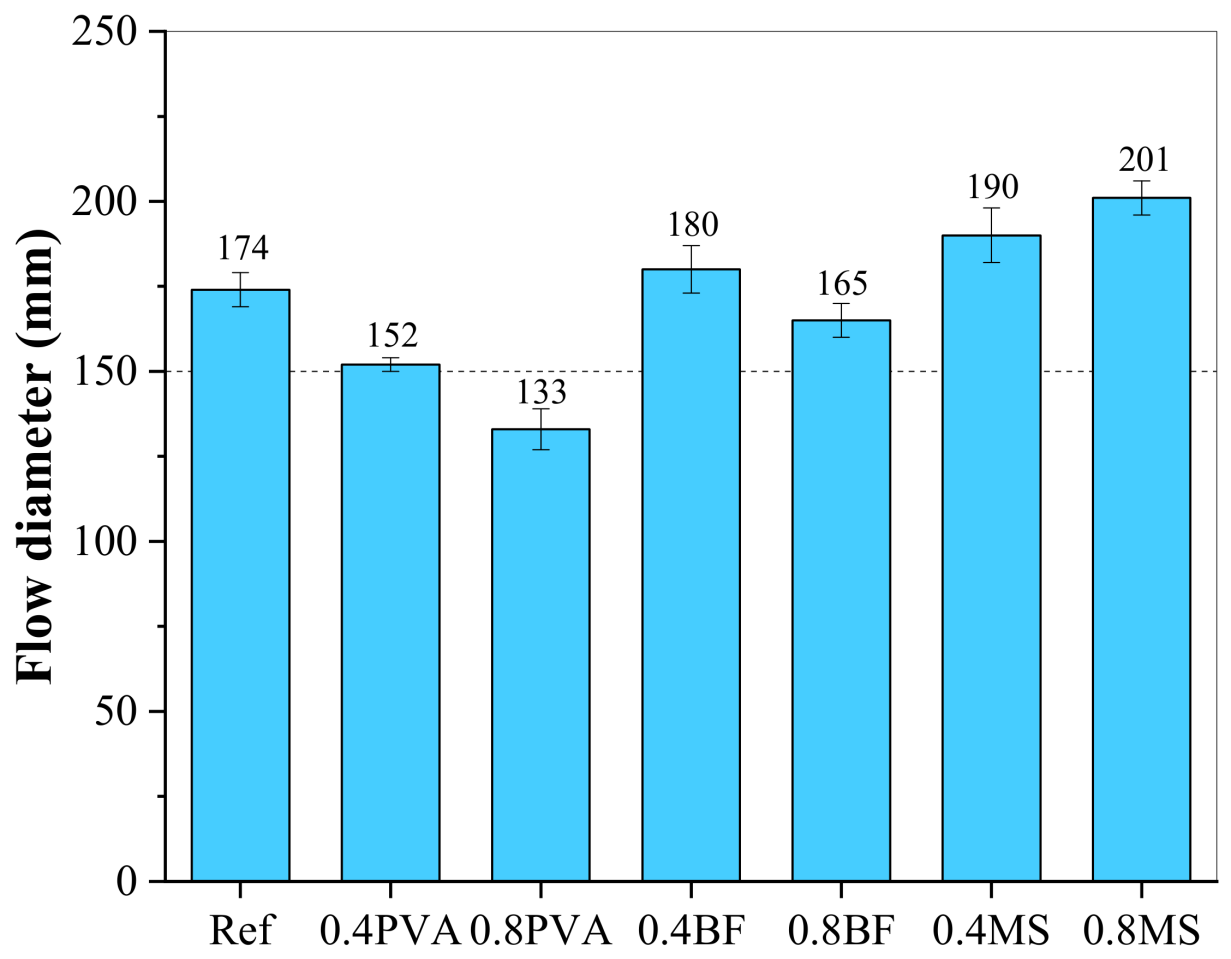
Flowability of geopolymer mortar mixtures containing different fiber types and contents.

The workability of the geopolymer mortar mixtures was evaluated using the flow table test, with the obtained results summarized in Table 6 and graphically presented in Figure 5.

The reference mixture exhibited a flowability of 174 mm, indicating adequate workability for casting and compaction. PVA fibers caused the greatest reduction in flowability, where the flow values decreased to 152 mm and 133 mm for fiber contents of 0.4% and 0.8%, respectively. This behavior is commonly attributed to the hydrophilic nature of PVA fibers and their high surface area, which increases water demand and reduces the mobility of the fresh mixture. In contrast, micro-steel fiber mixtures showed the highest flowability, reaching 190 mm and 201 mm for 0.4% and 0.8% fiber contents, respectively. Basalt fibers produced intermediate values of 180 mm and 165 mm. Overall, the results indicate that fiber type and dosage significantly influence the fresh-state workability of one-part geopolymer mortars.

### 3.2. Mechanical Properties

#### 3.2.1. Flexural Strength

**Figure 6:**
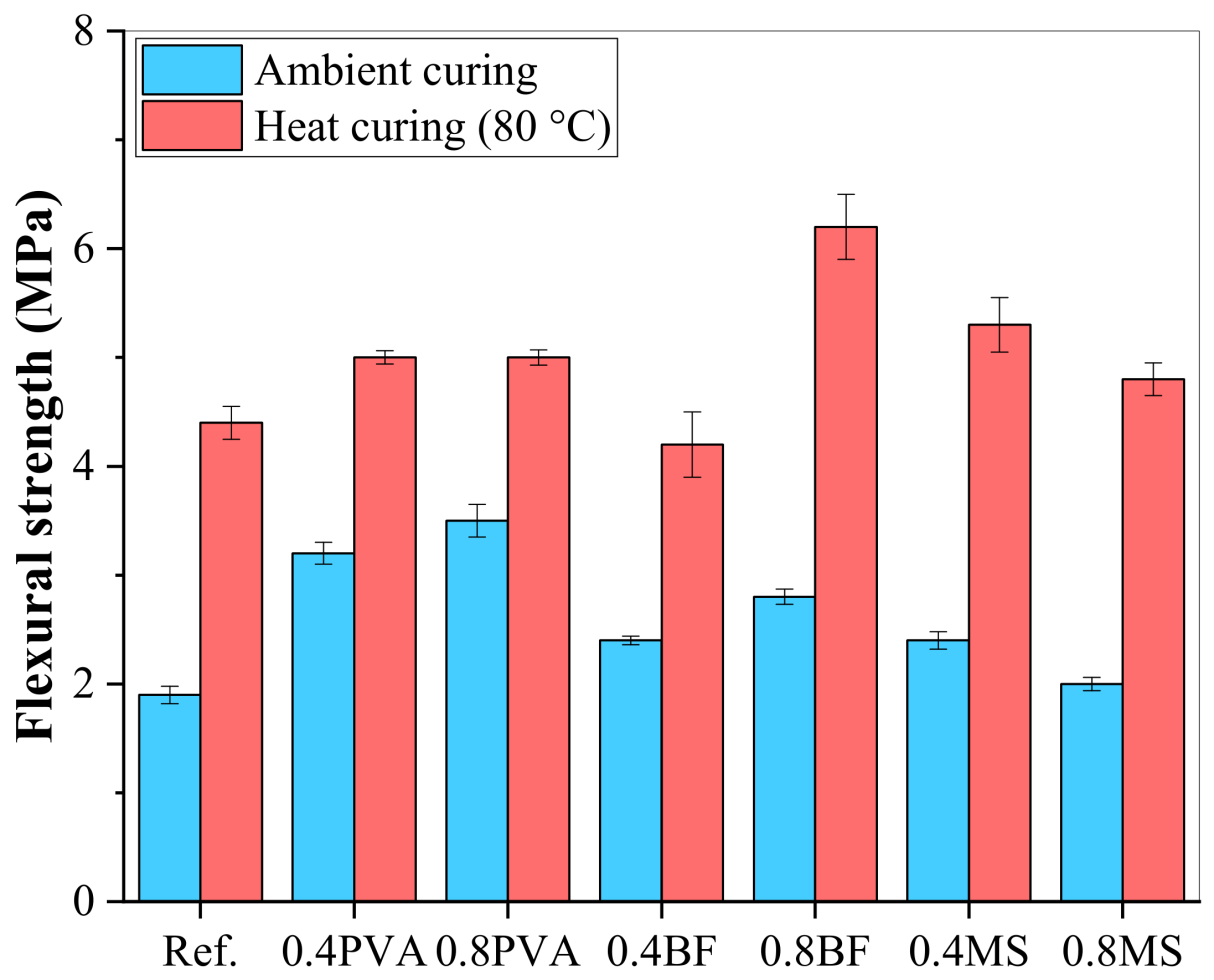
Flexural strength of geopolymer mortars after 7 days under ambient and heat curing conditions.

**Figure 7:**
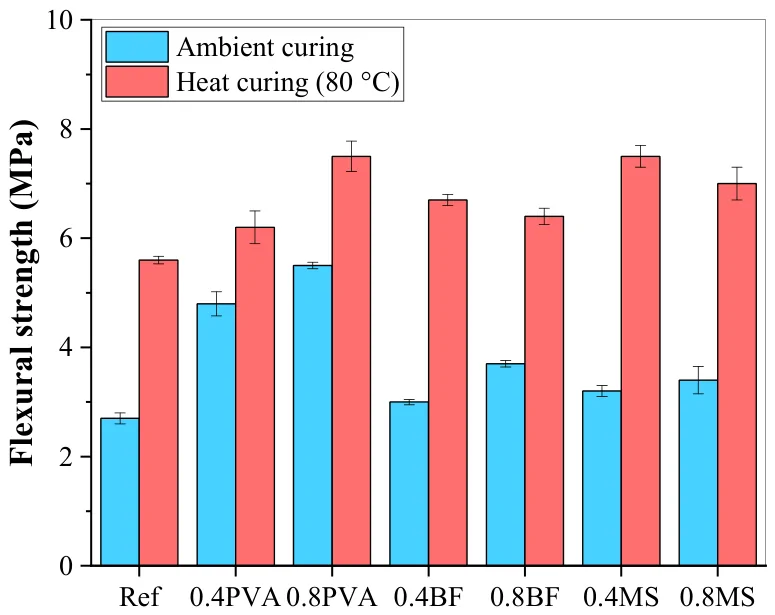
Flexural strength of geopolymer mortars after 28 days under ambient and heat curing conditions.

The flexural strength results obtained after 7 and 28 days are summarized in Table 7 and Table 8, respectively, and graphically presented in Figure 6 and Figure 7.

After 7 days of ambient curing, the reference mixture exhibited a flexural strength of 1.9 MPa, whereas the addition of fibers increased the flexural strength values to between 2.0 and 3.5 MPa. The highest value was recorded for the 0.8PVA mixture (3.5 MPa). Under heat curing at 80 °C, the flexural strength increased significantly, reaching 4.4 MPa for the reference mixture and up to 6.2 MPa for fiber-reinforced mixtures.

After 28 days, additional strength development was observed for all mixtures. The flexural strength ranged between 2.7 MPa and 7.5 MPa, with the highest values recorded for mixtures containing PVA and micro-steel fibers. The improvement in flexural strength can be attributed to the crack-bridging effect of fibers, which delays crack propagation and improves the tensile resistance of the matrix.

#### 3.2.2. Compressive Strength

**Figure 8:**
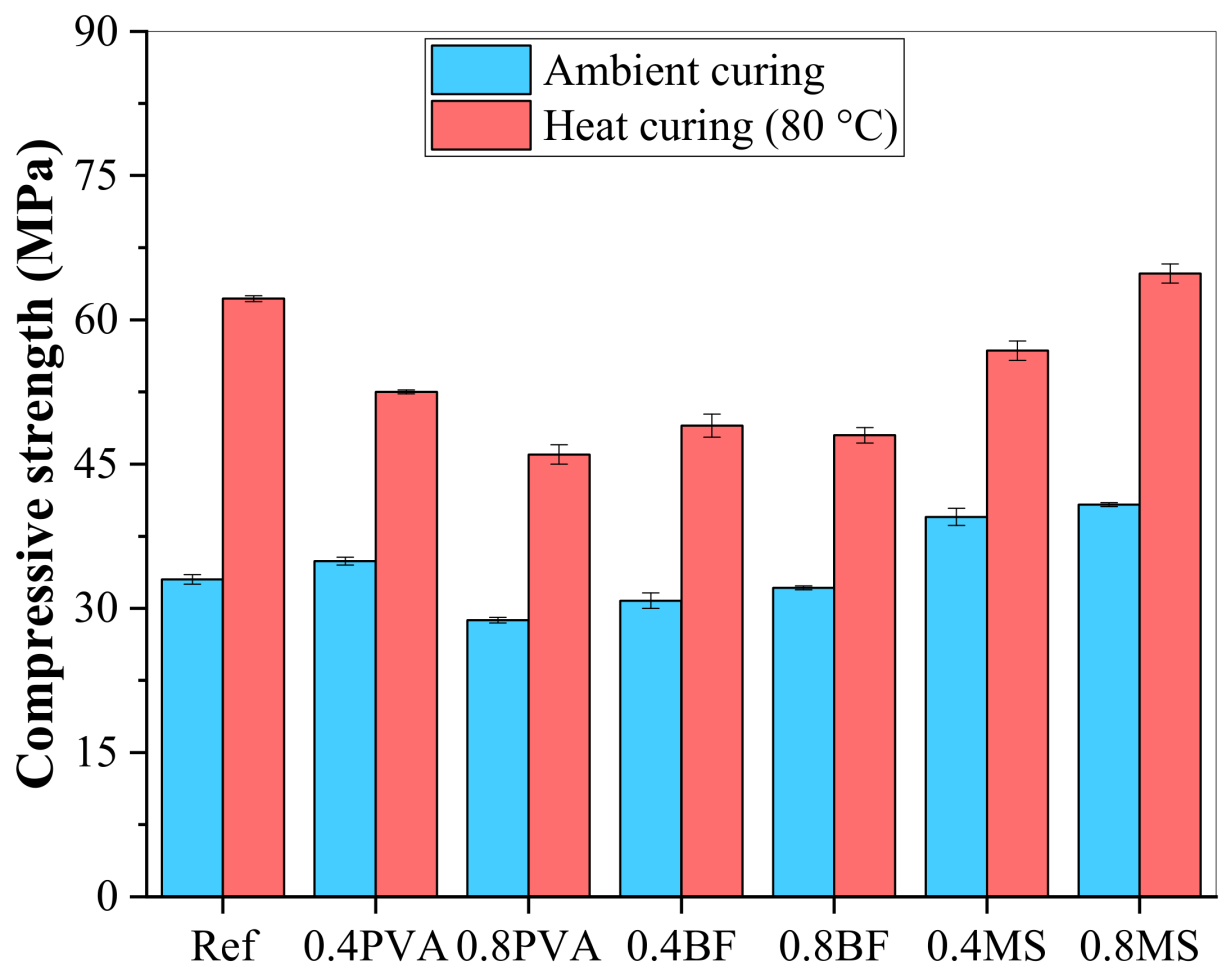
Compressive strength of geopolymer mortars after 7 days under ambient and heat curing conditions.

**Figure 9:**
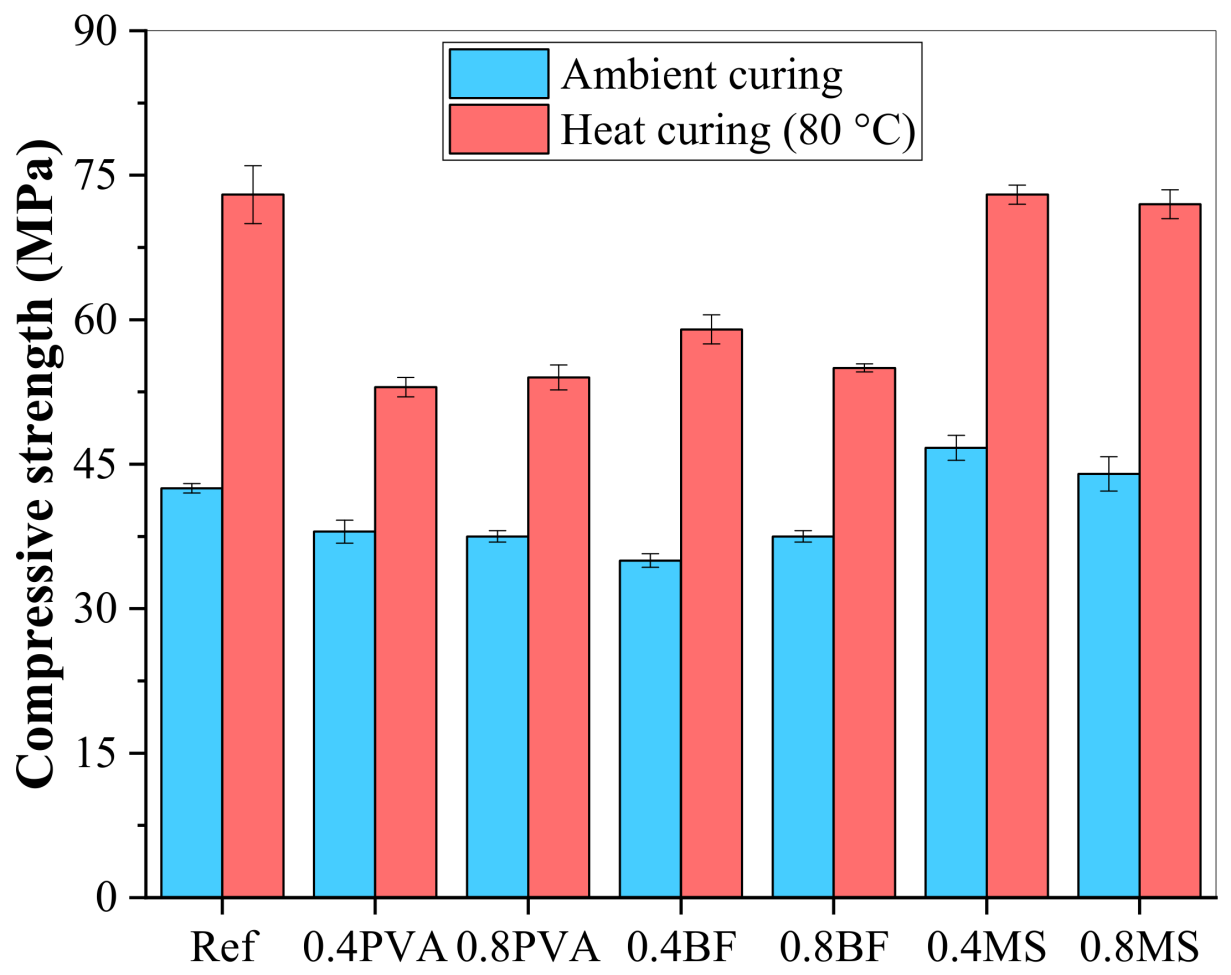
Compressive strength of geopolymer mortars after 28 days under ambient and heat curing conditions.

The compressive strength results obtained after 7 days are presented in Table 9, while the results after 28 days are summarized in Table 10. The corresponding results are graphically illustrated in Figure 8 and Figure 9 for the 7-day and 28-day curing periods, respectively.

After 7 days of ambient curing, the compressive strength ranged between 28.8 MPa and 40.8 MPa, with the highest value recorded for the 0.8MS mixture. Heat curing significantly enhanced strength development, where the compressive strength increased to 62.2 MPa for the reference mixture and reached 64.8 MPa for the 0.8MS mixture.

After 28 days, the compressive strength ranged between 35 MPa and 73 MPa. The highest values were again observed for mixtures containing micro-steel fibers, demonstrating their positive influence on load transfer and crack arrest.

#### 3.2.3. Splitting Tensile Strength

**Figure 10:**
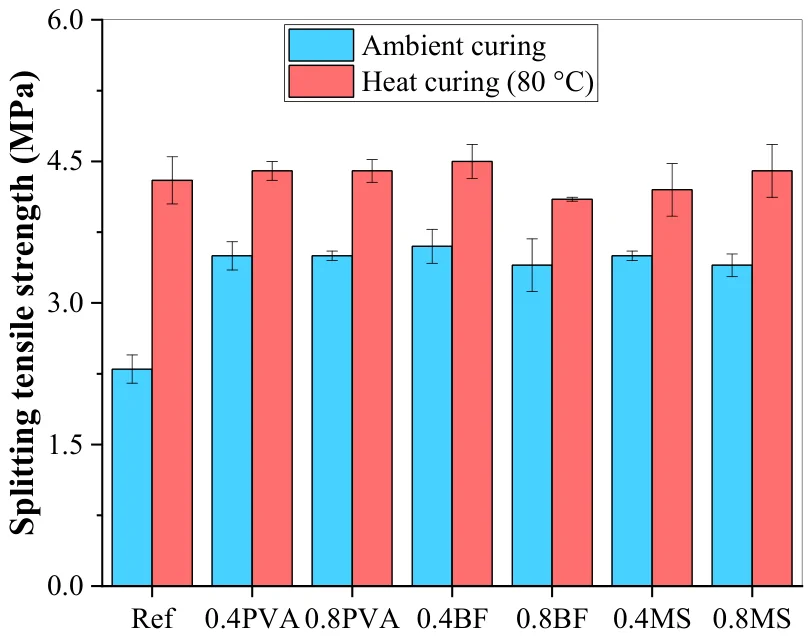
Splitting tensile strength of geopolymer mortars under ambient and heat curing conditions.

The splitting tensile strength results of the geopolymer mortars under both curing conditions are presented in Table 11. The corresponding results are graphically presented in Figure 10.

Splitting tensile strength increased from 2.3 MPa to 3.6 MPa with the fiber reinforcement under ambient curing. The highest ambient value was 3.6 MPa for 0.4BF. The reference mixture exhibited a splitting tensile strength of 2.3 MPa under ambient curing, which increased to 4.3 MPa under heat curing. Under heat curing, all mixtures fell within a narrow range of 4.1–4.5 MPa, with 0.4BF again giving the highest measured value. The data therefore support a general fiber-bridging benefit and improved the tensile behavior of geopolymer mortars under ambient curing, whereas differences among fiber types under heat curing were insignificant.

### 3.3. Ultrasonic Pulse Velocity (UPV)

**Figure 11:**
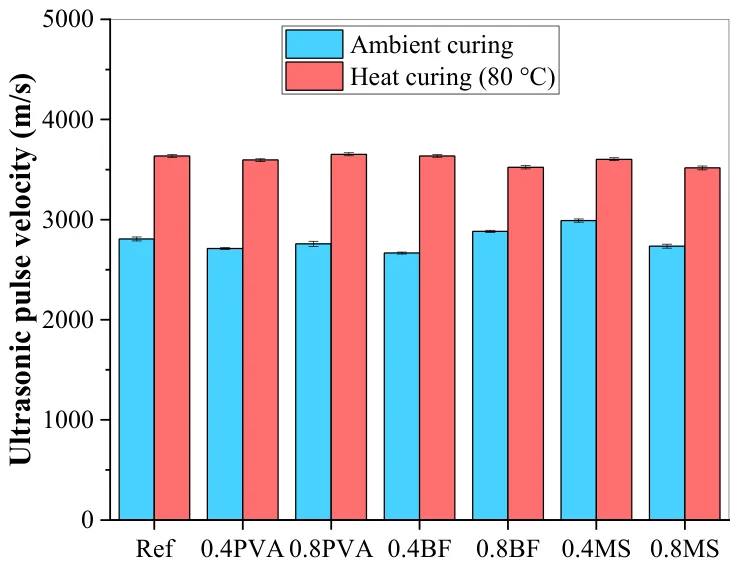
Ultrasonic pulse velocity of geopolymer mortar mixtures (28-day values).

Ultrasonic pulse velocity (UPV) measurements were performed to assess the internal quality and structural integrity of the developed geopolymer mortars. The 28-day UPV values of the geopolymer mortar mixtures under ambient and heat-curing conditions are graphically presented in Figure 11. The measured UPV values varied from 2666.7 to 3653.0 m/s, reflecting differences in matrix compactness and internal homogeneity among the investigated mixtures. In general, specimens cured under heat exhibited consistently higher UPV values than those cured under ambient conditions, indicating that elevated-temperature curing promoted a denser and more continuous internal structure. Among all mixtures, the 0.8PVA specimen under heat curing recorded the highest UPV value of 3653.0 m/s.

**Figure 12:**
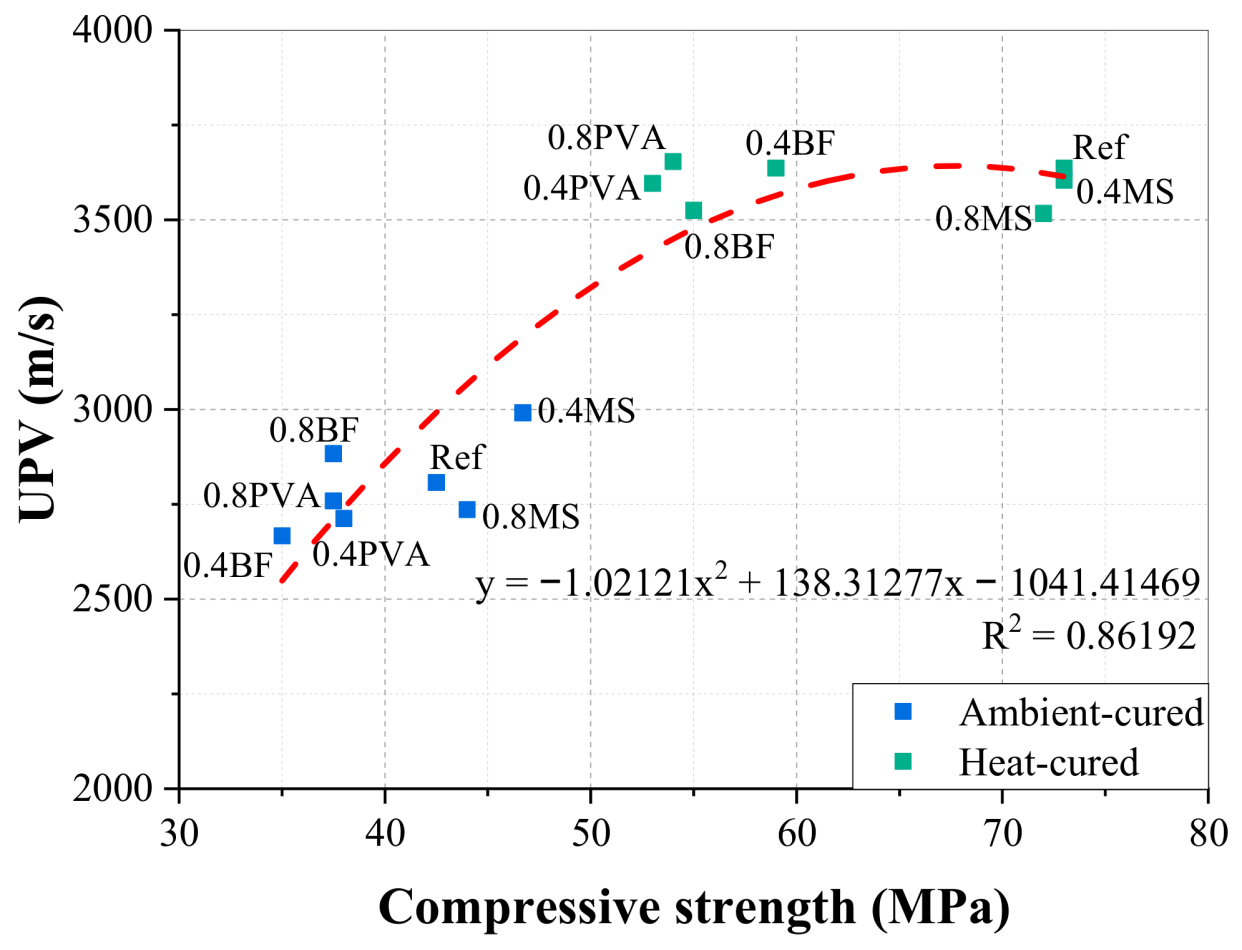
Relationship between UPV and 28-day compressive strength of mortars.

Figure 12 shows a clear nonlinear relationship between the 28-day compressive strength and UPV values. The quadratic regression provided a good fit to the experimental data, with an R^2^ value of 0.862. Ambient-cured mixtures were located mainly within the lower strength and UPV ranges, whereas heat-cured mixtures shifted toward higher values for both parameters. The slope of the fitted curve decreased beyond approximately 60 MPa, indicating that further increases in compressive strength were accompanied by relatively limited changes in UPV. Since the data formed two curing-related clusters and each point represents a mixture-level mean value, the regression should be regarded as a descriptive relationship rather than a general predictive model. Nevertheless, within the investigated composition range, UPV appears to be a useful nondestructive parameter for comparing mechanical development under different curing conditions.

### 3.4. Böhme Abrasion Resistance

**Figure 13:**
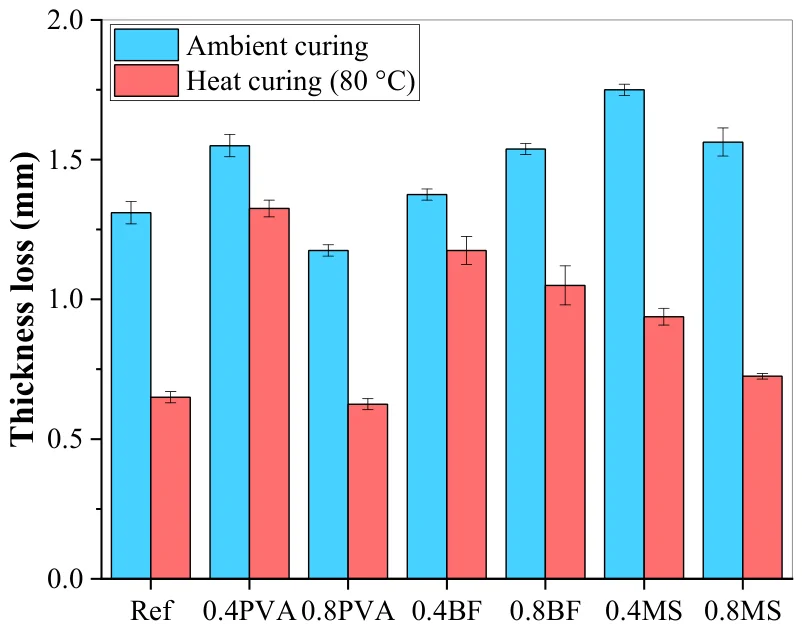
Thickness loss of geopolymer mortar mixtures—Böhme abrasion test under ambient and heat-curing conditions.

**Figure 14:**
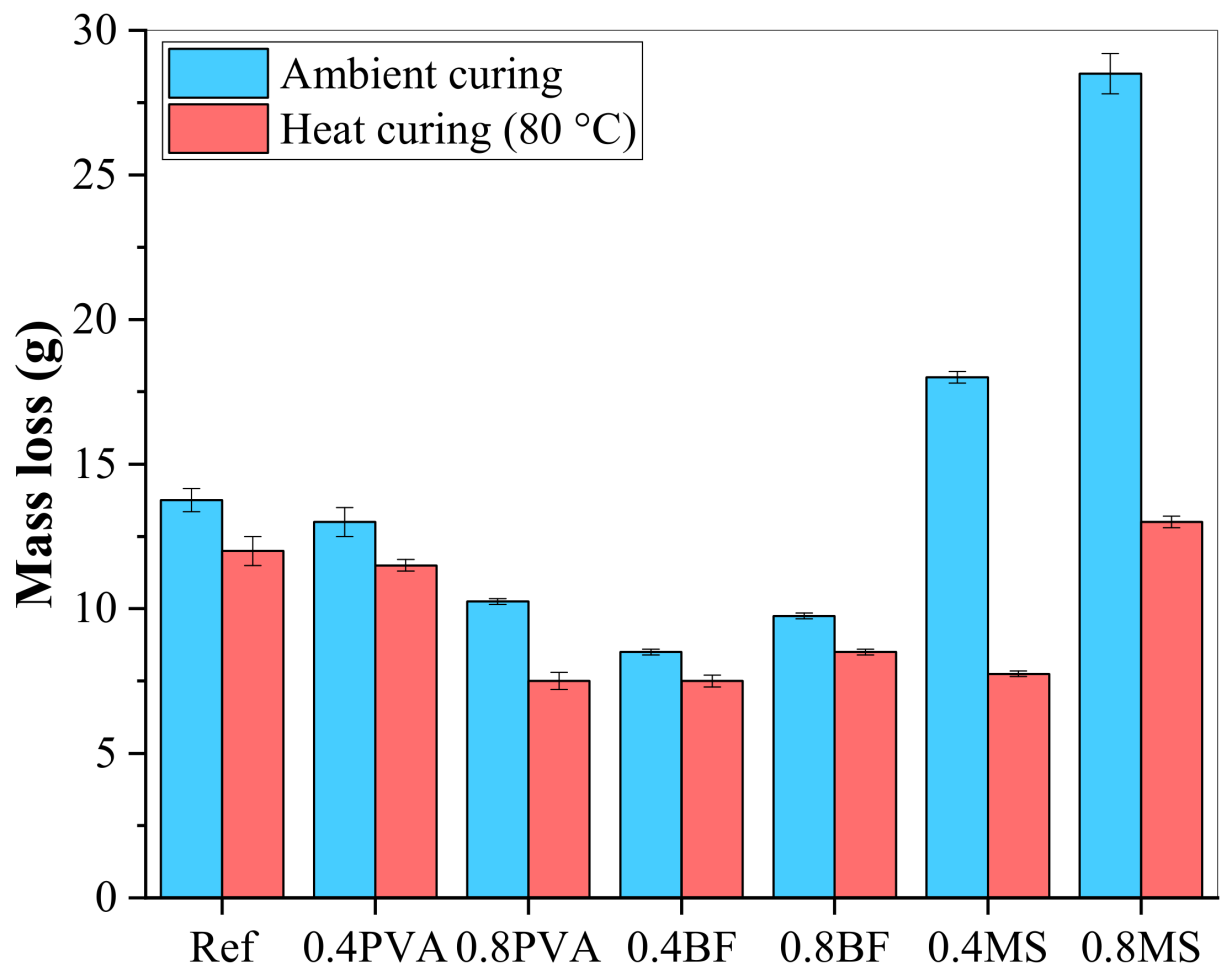
Mass loss of geopolymer mortar mixtures—Böhme abrasion test under ambient and heat-curing conditions.

The abrasion resistance of the developed geopolymer mortar mixtures was evaluated using the Böhme abrasion test under both ambient and heat-curing conditions. The calculated mass loss and thickness loss values are presented in Table 12 and Table 13. The thickness loss and mass loss results obtained from the Böhme abrasion test under ambient and heat-curing conditions are graphically presented in Figure 13 and Figure 14, respectively.

Thickness loss was used as the primary abrasion indicator. For the ambient-cured specimens, the thickness loss values ranged from 1.18 to 1.75 mm. The 0.8PVA specimen exhibited the lowest thickness loss (1.18 mm), indicating the highest abrasion resistance under ambient curing. For the heat-cured specimens, the abrasion resistance generally improved, with thickness loss values ranging from 0.63 to 1.33 mm. The 0.8PVA mixture again showed the best performance, with the lowest thickness loss of 0.63 mm. However, the reference was very close to 0.8PVA with 0.65 mm. Mass loss did not always give the same ranking: for example, ambient-cured 0.8MS had the largest mass loss (28.5 g) but not the largest thickness loss. The divergence is plausible because mass loss responds to density and discrete fragment detachment, while thickness loss reflects average surface recession. Localized spalling, exposure or pull-out of fibers, and nonuniform wear can therefore change mass without producing a proportional change in mean thickness.

These results indicate that elevated-temperature curing contributed to the formation of a denser and more cohesive matrix, thereby improving resistance to mechanical surface wear. Moreover, the superior performance of the PVA-reinforced mixtures at 0.8% dosage suggests that the presence of well-dispersed fibers contributed to limiting surface degradation.

### 3.5. Water Absorption and Capillary Water Uptake

**Figure 15:**
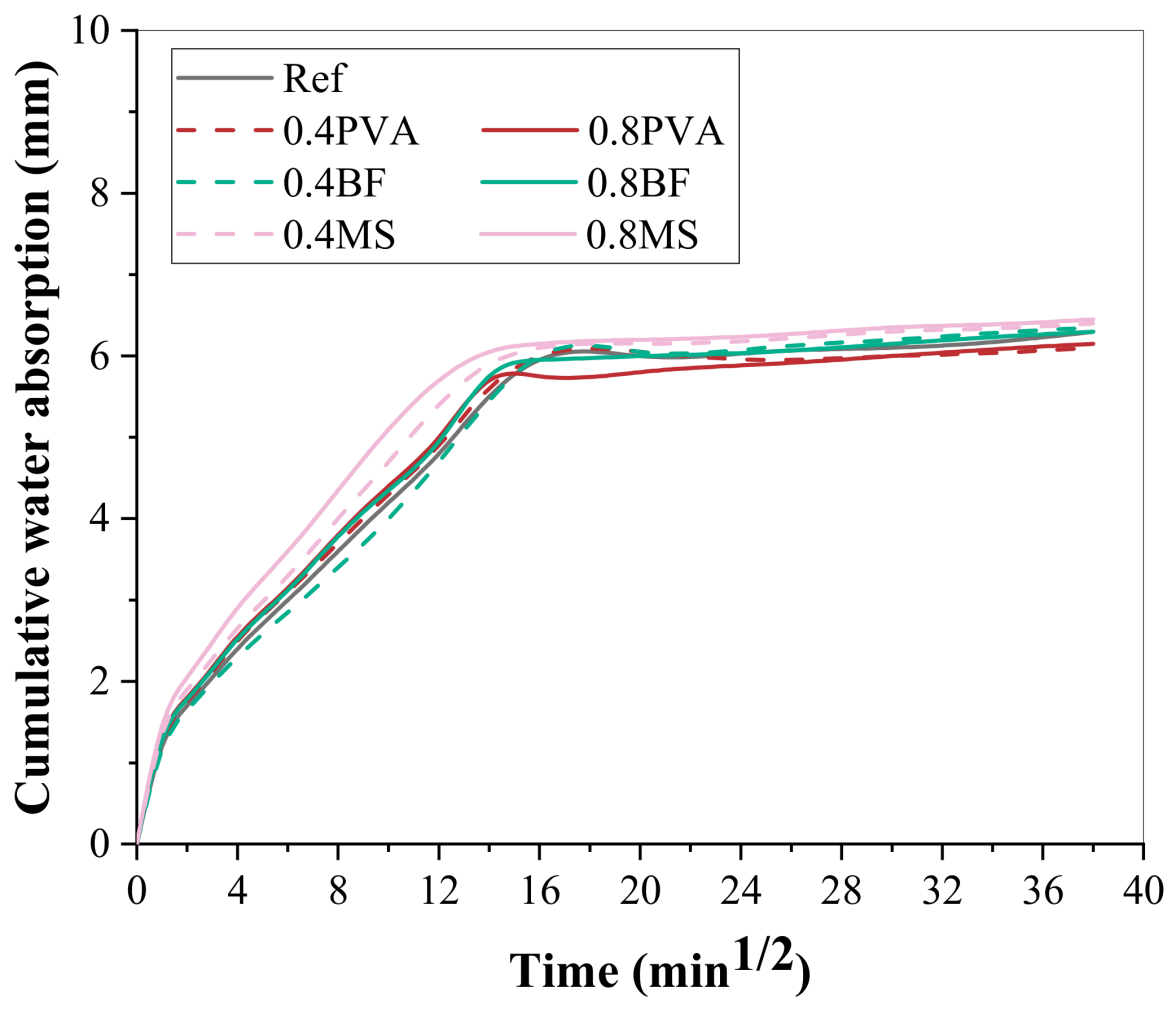
Capillary water absorption curves of geopolymer mortars under ambient curing conditions.

**Figure 16:**
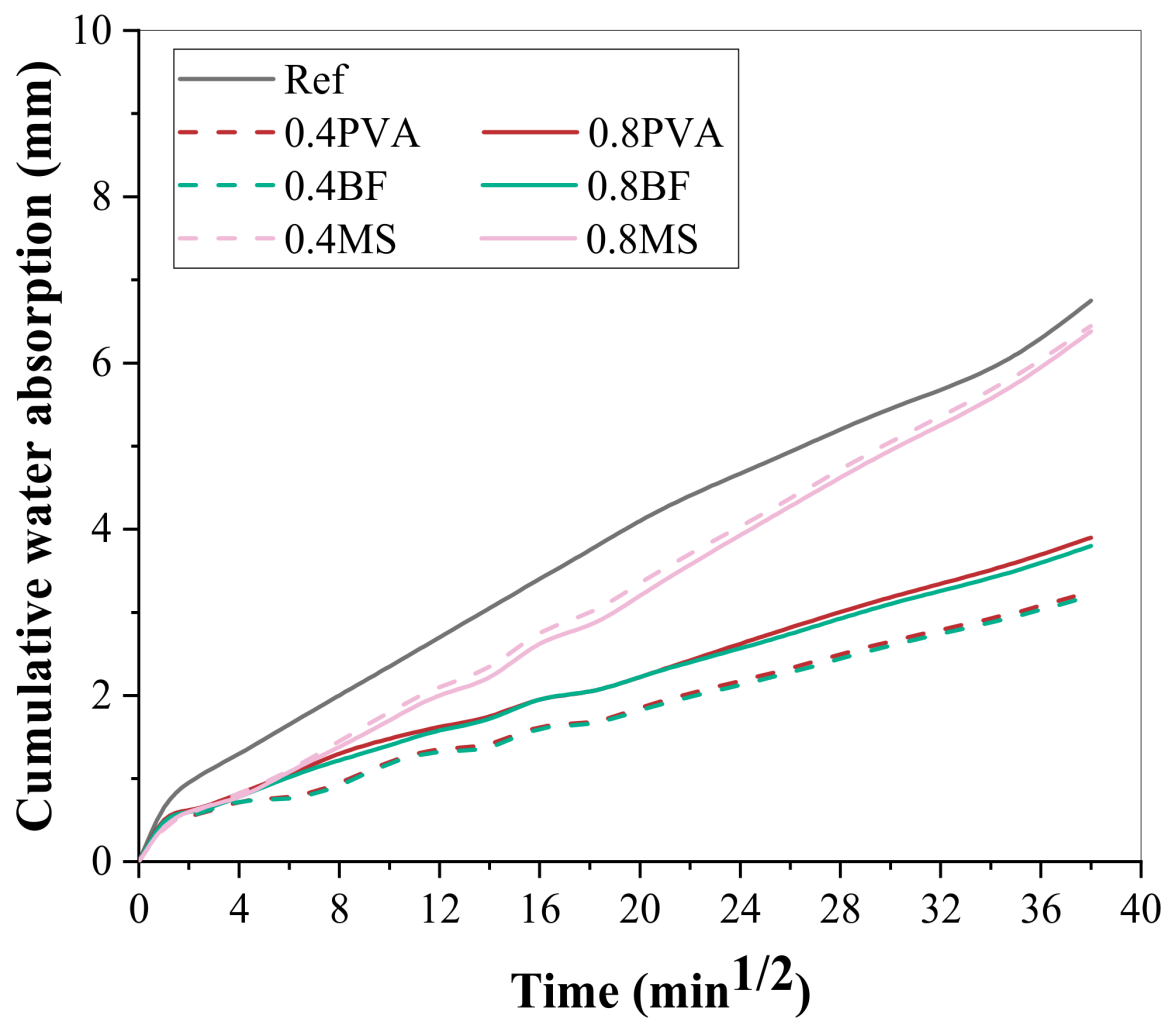
Capillary water absorption curves of geopolymer mortars under heat curing (80 °C).

The capillary water absorption behavior of the geopolymer mortars was evaluated by measuring the increase in specimen mass during water exposure under two curing regimes. Water absorption after 24 h was calculated according to:

*WA*_24h_ (%) = [(*M*_24h_ − *M*_0_)/*M*_0_] × 100

where WA_24h_ is the water absorption after 24 h (%), M_0_ is the initial dry mass (g), and M_24h_ is the mass after 24 h of water exposure (g). The calculated 24 h water absorption values are summarized in Table 14. The cumulative capillary water absorption curves of the geopolymer mortar mixtures under ambient and heat-curing conditions are presented in Figure 15 and Figure 16, respectively.

This behavior may be attributed to the influence of the fiber–matrix interfacial transition zone, which can affect long-term water transport through the matrix.

The 24 h water-absorption response depended strongly on fiber type. Heat curing reduced absorption by 36.3–47.4% for PVA mixtures and by 41.9–49.2% for basalt mixtures. In contrast, the reference increased from 4.18% to 4.40%, and the micro-steel mixtures remained high at 4.42–4.43%, representing slight increases of 2.3–3.5%. Heat curing therefore did not produce a uniform improvement in this indicator; the benefit was concentrated in the PVA- and basalt-reinforced mixtures. The reason for this behavior may be that heat curing densified the matrix and fiber–matrix interface in the PVA- and basalt-fiber mixtures, thereby reducing connected capillary pores. In the micro-steel-fiber mixtures, weaker interfacial zones, local voids, or a lower fiber count may have limited this beneficial effect.

**Figure 17:**
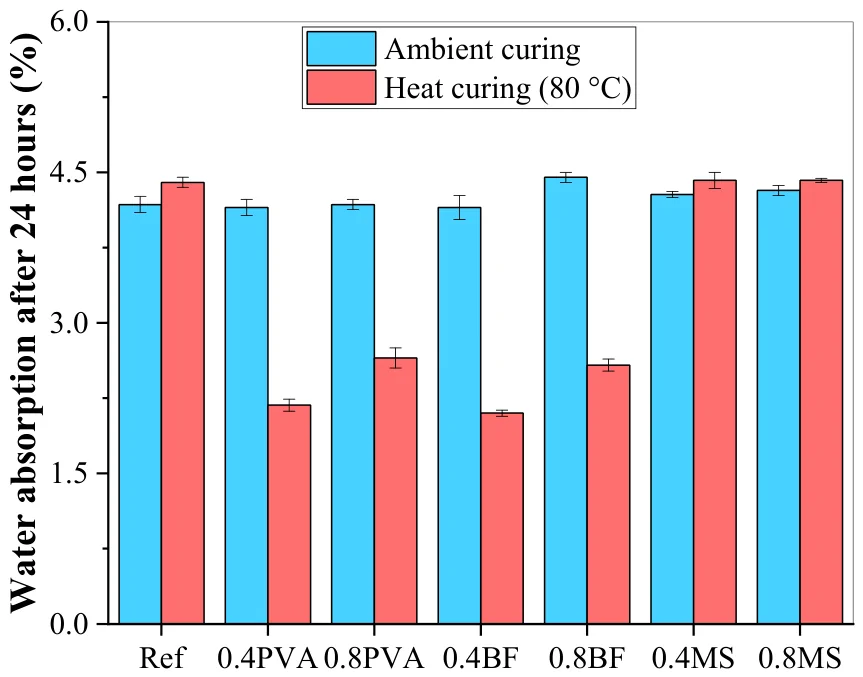
Water absorption of geopolymer mortars after 24 h under ambient and heat curing conditions.

**Figure 18:**
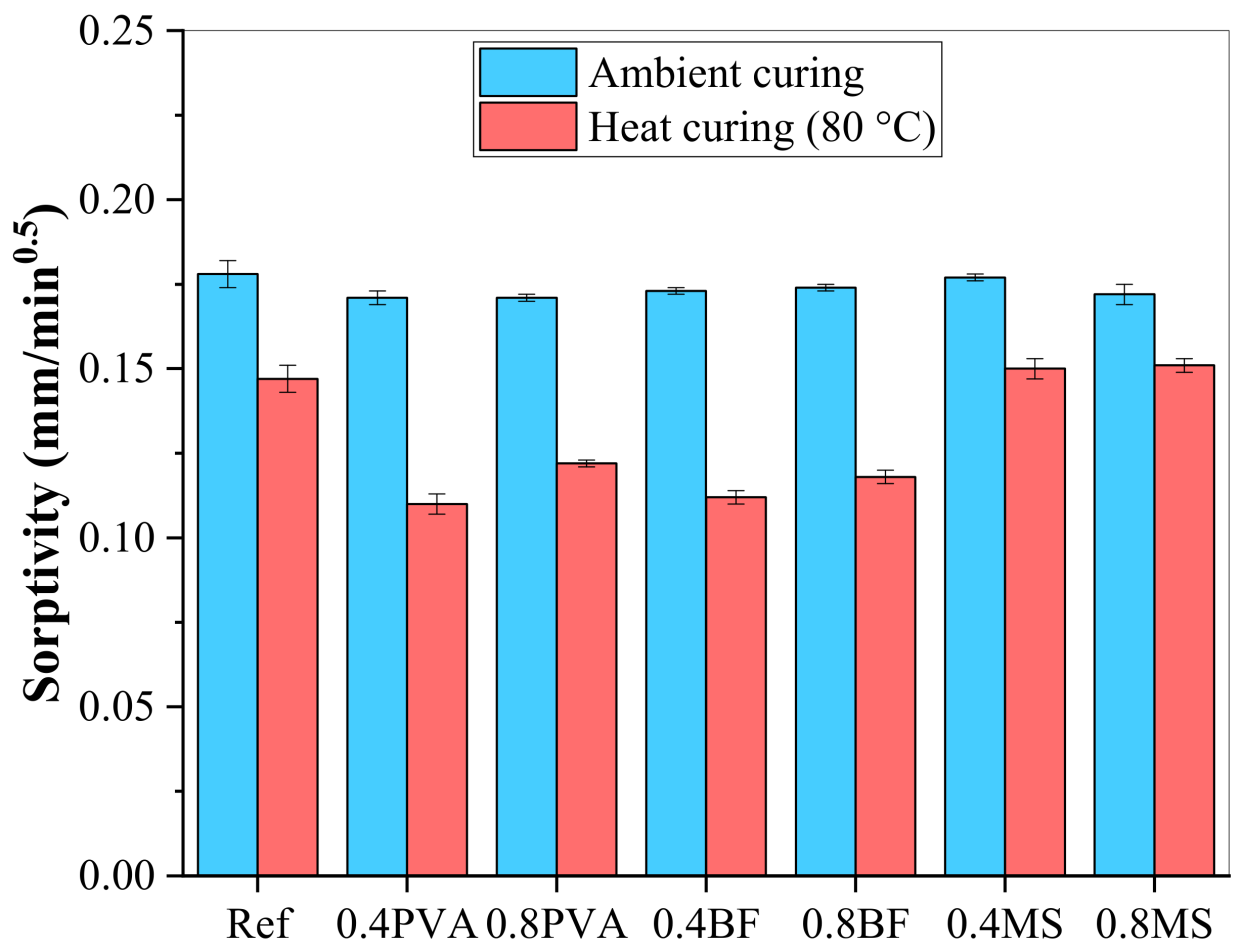
Sorptivity of geopolymer mortars under ambient and heat curing conditions.

To further evaluate the capillary transport properties of the geopolymer mortars, the sorptivity coefficient was determined from the relationship between cumulative water absorption and the square root of time. Sorptivity was calculated according to I = Δm/(Aρ) and S = dI/d√t. The calculated sorptivity values are presented in Table 15. The 24 h water absorption and sorptivity results of the geopolymer mortar mixtures under ambient and heat-curing conditions are graphically presented in Figure 17 and Figure 18, respectively.

The sorptivity results confirm the trends observed in the water absorption data. Specimens subjected to heat curing exhibited lower sorptivity values compared with ambient-cured specimens, indicating improved resistance to capillary water transport due to a more compact geopolymer matrix. Among the tested mixtures, PVA and basalt fiber-reinforced mortars exhibited the lowest sorptivity values, suggesting that these fibers contribute to better crack control and pore refinement. In contrast, mixtures containing micro-steel fibers showed slightly higher sorptivity values, which may be related to the characteristics of the fiber–matrix interface and its influence on long-term water transport.

### 3.6. Mineralogical, Spectroscopic, and Microstructural Characterization

#### 3.6.1. XRD Analysis

**Figure 19:**
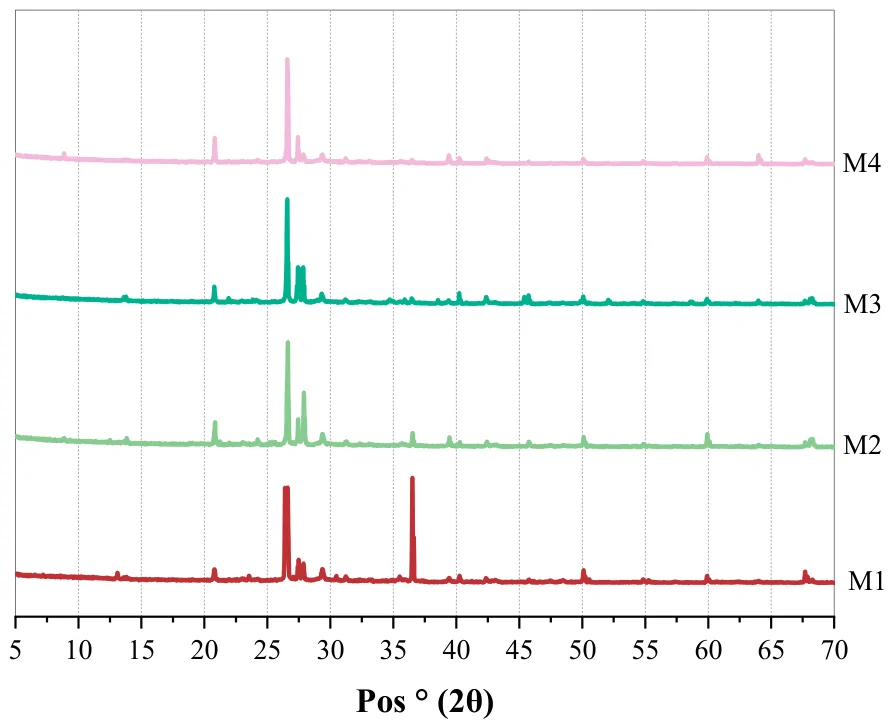
XRD patterns of heat-cured geopolymer mortars. M1 = Ref; M2 = 0.8PVA; M3 = 0.8BF; M4 = 0.8MS.

The XRD patterns of the heat-cured one-part geopolymer mortars (M1–M4) are presented in Figure 19. The XRD analysis was used for qualitative comparison of the diffraction features. The mixtures exhibited broadly comparable diffraction profiles, with prominent reflections at approximately 20.8°, 26.6°, 27.4–27.9°, and 29.3° 2θ. The intense reflection at approximately 26.6° 2θ, together with the reflection near 20.8° 2θ, is consistent with residual quartz originating from the aluminosilicate precursors. However, the reflections within the 27–30° 2θ region could not be assigned unambiguously because several precursor-derived (e.g., residual quartz, mullite) and carbonate-containing (e.g., calcite) crystalline phases may exhibit overlapping reflections in this interval. The reflection near 29.3° 2θ may be associated with calcite or another carbonate-containing constituent, although a definitive distinction cannot be made from peak positions alone. Minor reflections observed throughout the patterns may also be compatible with Mg- and Na- containing crystalline phases that can occur in alkali-activated systems, including hydrotalcite, brucite, hydrogarnet or gaylussite-related phases. Nevertheless, these phases cannot be conclusively identified from peak positions alone because of the substantial overlap among their characteristic reflections. The persistence of residual crystalline constituents inherited from the original raw materials, superimposed on the geopolymer reaction products. This behavior is typical of alkali-activated and geopolymer systems, where the binder phase is predominantly poorly crystalline or amorphous, while some precursor-derived crystalline phases remain partially unreacted [43,44].

Although slight differences in peak intensity were observed among the mixtures, the overall diffraction profiles were similar. The M3 specimen showed comparatively stronger reflections in some characteristic regions, particularly near 26.6°, 27.9°, and 29.3° 2θ. However, these intensity differences may reflect variations in the relative abundance of residual crystalline constituents, specimen heterogeneity, or preferred orientation and should not be interpreted directly as differences in reaction degree. No additional distinct reflections attributable to fiber addition were observed within the detection limits of the XRD analysis. Thus, fiber incorporation did not appear to produce readily detectable changes in the crystalline phase assemblage.

#### 3.6.2. FTIR Analysis

**Figure 20:**
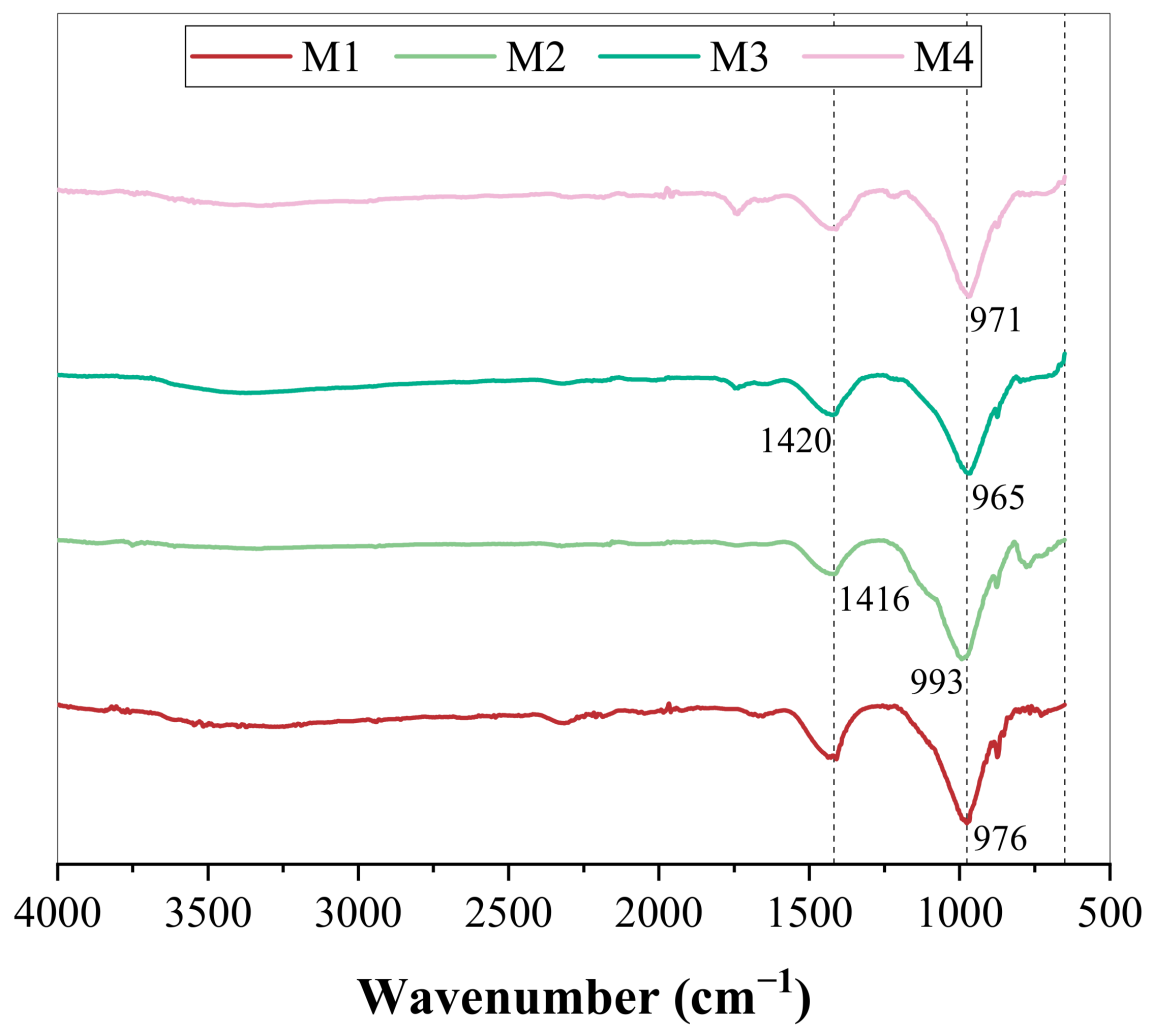
FTIR spectra of heat-cured geopolymer mortars M1–M4. The main absorption bands associated with the geopolymeric aluminosilicate network and carbonate-containing phases are indicated.

The FTIR spectra of the heat-cured geopolymer mortars (M1–M4) are presented in Figure 20, and the principal absorption bands identified for each specimen are summarized in Table 16. In all investigated specimens, the dominant absorption band was observed in the range of approximately 965–993 cm^−1^. This band is generally assigned to the asymmetric stretching vibration of Si–O–T bonds (T = Si or Al) in the geopolymeric aluminosilicate framework. The presence of this band in all mixtures confirms the formation of a geopolymer-type reaction network in the developed one-part mortar system [44,45].

Such band shifts are commonly associated with variations in the degree of polymerization, local Al substitution, and the interaction between the binder matrix and incorporated fibers [45]. Additional bands were identified in the spectra of M2 and M3 at 1416 cm^−1^ and 142 cm^−1^, respectively. These bands are typically associated with carbonate-related vibrations. This is consistent with the use of sodium carbonate in the solid alkaline activator and with the known tendency of alkali-activated systems to exhibit carbonate-related absorption bands [44].

#### 3.6.3. SEM/EDS Analysis

**Figure 21:**
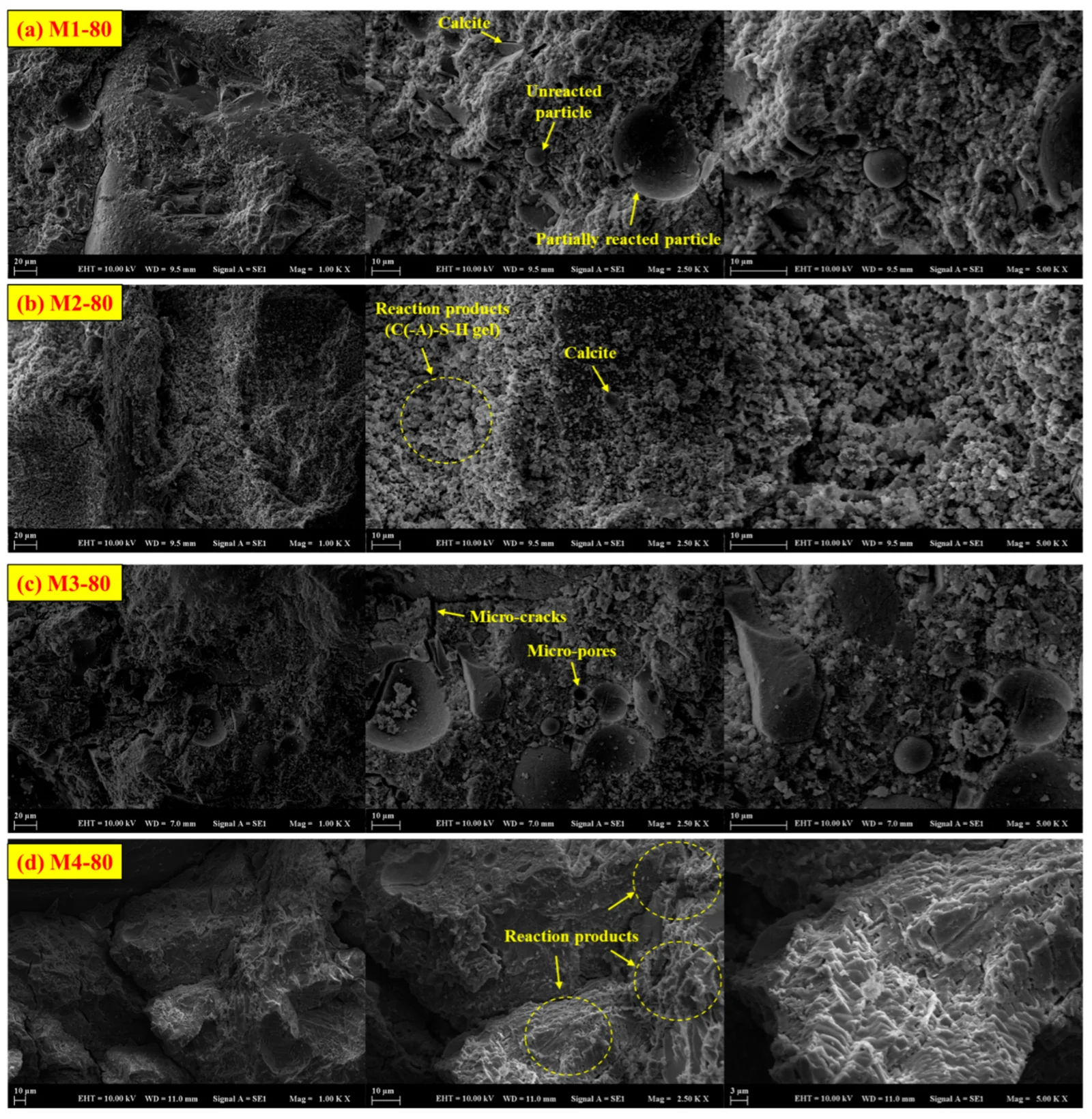
SEM micrographs of (**a**) M1-80; (**b**) M2-80; (**c**) M3-80; (**d**) M4-80mixtures obtained at magnifications of ×1000, ×2500, and ×5000.

Representative SEM micrographs and corresponding EDS results are presented in Figure 21. The SEM observations revealed a relatively dense and heterogeneous geopolymer matrix containing reacted particles together with some unreacted or partially reacted particles embedded within the matrix, especially in the heat-cured specimens. Such a morphology is commonly observed in one-part geopolymer systems, where the extent of dissolution and reaction may vary locally depending on precursor reactivity and curing conditions [43,44]. It should be noted that the current SEM investigation primarily focused on the overall bulk matrix morphology, compactness, and the distribution of reaction products under heat curing. Consequently, specific high-resolution imaging explicitly targeting the fiber–matrix interfacial transition zone (ITZ)—which is necessary to visually evaluate fiber pull-out, debonding, and localized mechanical interlocking mechanisms—was not captured. This lack of direct ITZ visualization constitutes a limitation of the present microstructural scope, and detailed interfacial characterization remains a primary objective for future studies.

**Figure 22:**
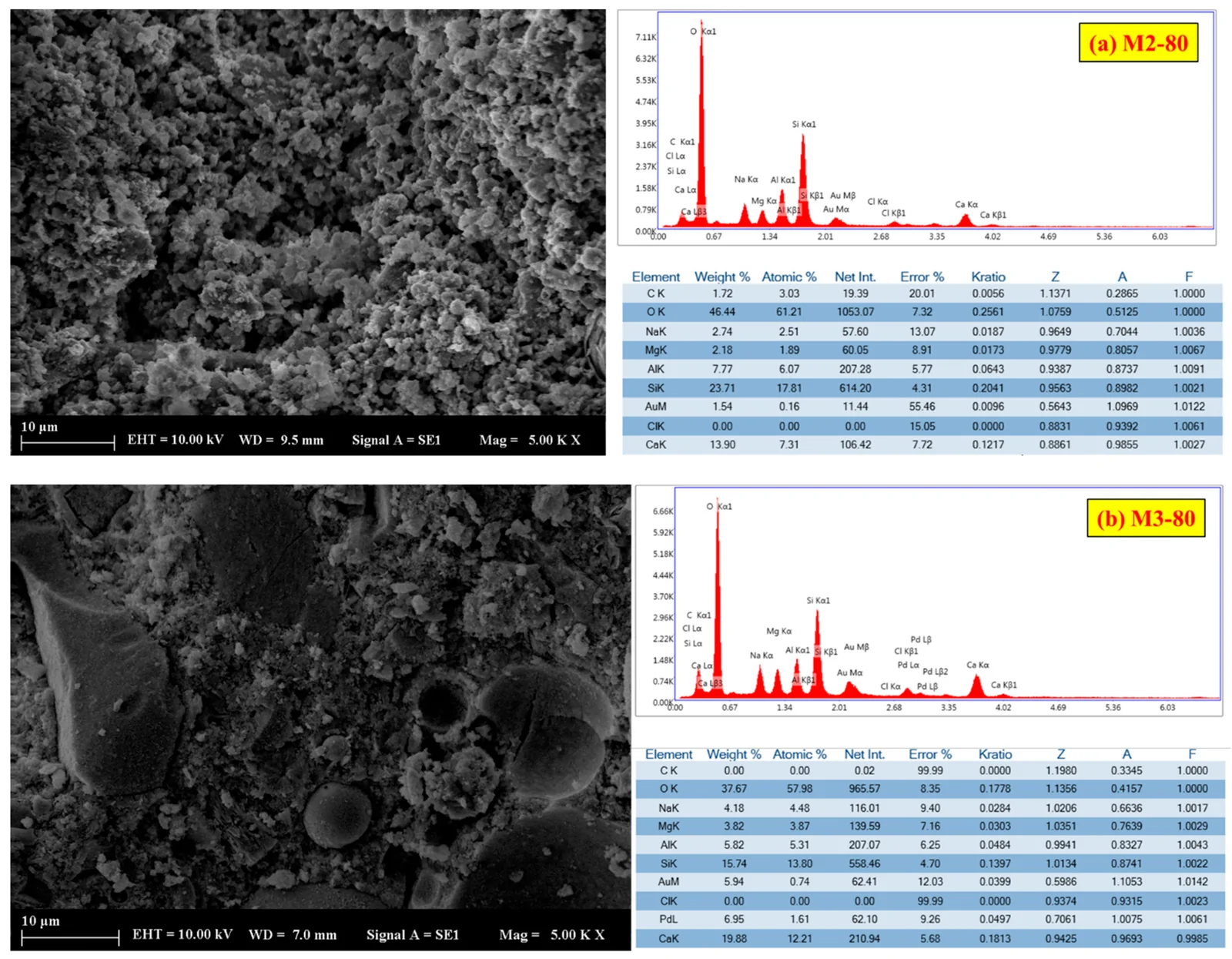
EDS spectra of (**a**) M2-80 and (**b**) M3-80 mixtures.

The EDS results presented in Figure 22 qualitatively confirmed the presence of the main elements expected in the investigated system, including Si, Al, Ca, Na, Mg, Fe and O. The coexistence of these elements supports the formation of a multi-component alkali-activated matrix. In particular, the presence of Si and Al together with significant Ca content, which reflects the role of slag in the development of a dense hardened matrix, suggests the formation of a hybrid reaction matrix consisting predominantly of calcium-aluminosilicate-hydrate (C(-A)-S-H) type gels together with Na-containing aluminosilicate reaction products. The detection of Mg is consistent with the incorporation of MgO and might indicate the formation of Mg-containing secondary phases (e.g., hydrotalcite, brucite); however, EDS alone cannot confirm the formation or role of specific Mg-containing secondary phases. The detection of Fe is also compatible with the incorporation of red mud in the precursor system. The elemental distribution obtained from the EDS analyses was generally consistent with the precursor composition and XRD and FTIR findings.

### 3.7. Life Cycle Assessment

#### 3.7.1. Comparison of Ambient and Heat-Curing Scenarios

**Figure 23:**
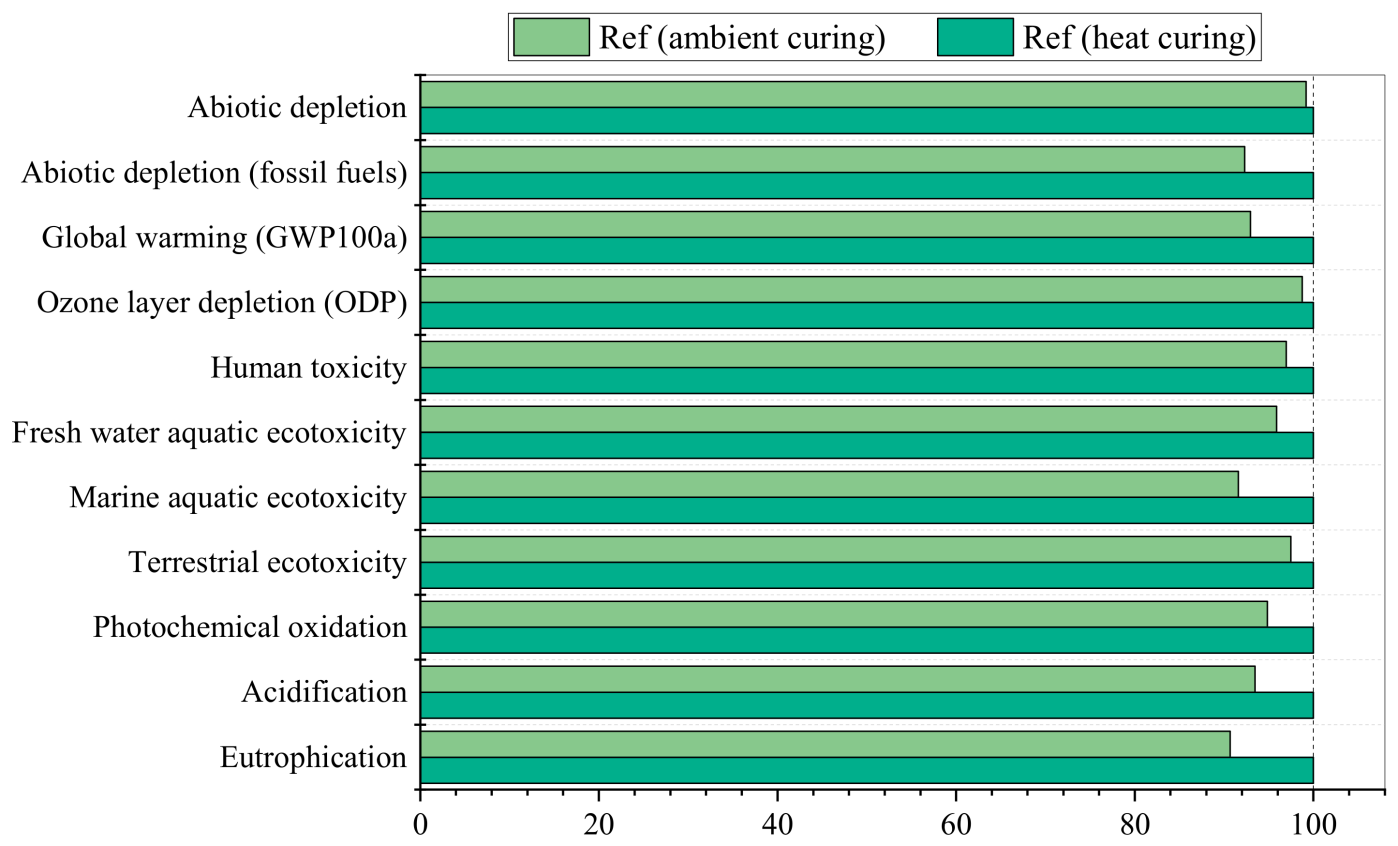
Percentage comparison of characterized environmental impacts for ambient-cured and heat-cured reference mixtures.

The characterized LCA results of the ambient-cured and heat-cured reference mixtures are presented in Table 17 and Figure 23. As expected, the heat-cured reference mixture exhibited higher environmental impacts in all selected CML-IA baseline impact categories due to the additional electricity input associated with oven curing at 80 °C for 24 h. However, the magnitude of increase varied across the impact categories, indicating that heat curing does not affect all environmental indicators equally.

Global warming potential (GWP100a) measures the contribution to climate change over a 100-year time horizon and is expressed in kg CO_2_ equivalent. The ambient-cured mixture had a total GWP of 267.37 kg CO_2_ eq, while the heat-cured mixture reached 287.67 kg CO_2_ eq. This represents a 7.60% increase. Although heat curing increased the carbon footprint, the increase remained below 10%, suggesting that the total GWP was still largely governed by the raw materials and activator-related inputs rather than curing electricity alone.

For resource-related indicators, the influence of heat curing remained comparatively limited. Abiotic depletion, which reflects the consumption of non-living mineral and elemental resources and is expressed in kg Sb equivalent, increased only slightly from 0.00238 kg Sb eq for the ambient-cured mixture to 0.00240 kg Sb eq for the heat-cured mixture. This corresponds to an increase of 0.84%, suggesting that the additional electricity demand associated with oven curing had only a minor contribution to mineral resource depletion. In this category, the overall impact appears to be governed primarily by the material-related burdens already present in the reference mixture. In contrast, abiotic depletion of fossil fuels showed a more pronounced increase, rising from 2726.44 MJ to 2953.53 MJ, corresponding to an 8.33% increase. This result is expected, as oven curing directly increases energy demand and therefore has a stronger effect on fossil energy resource consumption.

The effect of heat curing was less pronounced for ozone layer depletion. This impact category increased from 8.69 × 10^−6^ kg CFC-11 eq to 8.80 × 10^−6^ kg CFC-11 eq, corresponding to an increase of only 1.25%. The limited variation suggests that the electricity used for heat curing had a marginal influence on ozone-depleting emissions compared with the other life-cycle inputs considered in the reference mixture. For toxicity-related categories, the results showed moderate but category-dependent increases. Human toxicity increased from 324.29 kg 1,4-DB eq under ambient curing to 334.47 kg 1,4-DB eq under heat curing, corresponding to a 3.14% increase. This relatively limited change suggests that although electricity consumption contributed to the toxicity profile, the overall result remained mainly controlled by material production and supply-related processes. Freshwater aquatic ecotoxicity followed a similar trend, increasing from 238.88 kg 1,4-DB eq to 249.63 kg 1,4-DB eq, with a relative increase of 4.50%. Terrestrial ecotoxicity was also only moderately affected, increasing from 2.154 kg 1,4-DB eq to 2.210 kg 1,4-DB eq, corresponding to a 2.58% increase.

Among the toxicity-related categories, marine aquatic ecotoxicity displayed a comparatively stronger response to heat curing. The impact increased from 395,923.96 kg 1,4-DB eq for the ambient-cured reference mixture to 429,201.07 kg 1,4-DB eq for the heat-cured mixture, corresponding to an 8.40% increase. This makes marine aquatic ecotoxicity one of the more sensitive categories to the curing regime. The result indicates that the electricity input associated with oven curing contributed noticeably to marine ecotoxicity-related emissions, likely through upstream processes linked to electricity generation.

The remaining midpoint categories also confirmed the sensitivity of certain emission-related indicators to heat curing. Photochemical oxidation, expressed in kg C_2_H_4_ equivalent and associated with the formation potential of photochemical oxidants, increased from 0.0591 kg C_2_H_4_ eq to 0.0623 kg C_2_H_4_ eq, corresponding to a 5.41% increase. Acidification, which reflects the potential contribution of emissions such as SO_2_, NO_x_ and NH_3_ to acidifying effects in soil and water, increased from 1.199 kg SO_2_ eq to 1.283 kg SO_2_ eq, representing a 7.00% increase. These results indicate that the additional electricity demand for heat curing had a clear influence on air-emission-related impact categories.

Eutrophication exhibited the highest relative increase among all categories. The value rose from 0.456 kg PO_4_^3−^ eq for the ambient-cured mixture to 0.503 kg PO_4_^3-^ eq for the heat-cured mixture, corresponding to a 10.47% increase.

#### 3.7.2. Contribution Analysis of the Ambient-Cured Reference Mortar

**Figure 24:**
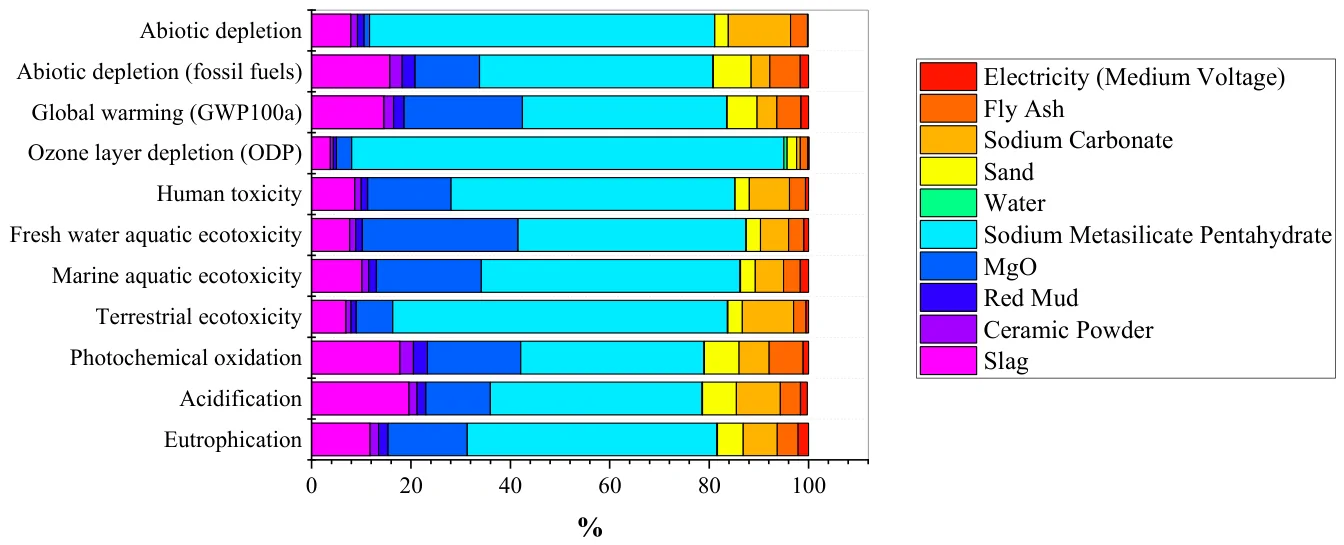
Relative contribution of materials and processes to the environmental impact categories of the ambient-cured reference mortar.

The contribution of each material and process to the characterized environmental impacts of the ambient-cured reference mixture is presented in Table 18 and Figure 24. Contribution analysis was carried out to identify the main environmental hotspots of the reference one-part geopolymer matrix before evaluating the additional burden introduced by heat curing. Across the selected CML-IA baseline categories, sodium metasilicate pentahydrate emerged as the principal contributor to the environmental profile of the mixture. Its dominance was particularly evident in GWP100a, resource depletion, ozone layer depletion and toxicity-related categories, while MgO, slag and sodium carbonate became relevant secondary contributors depending on the impact mechanism considered. By contrast, water, ceramic powder, red mud, fly ash and mixing electricity generally made only minor contributions to the total impacts.

Given its central relevance in the environmental assessment of cementitious and alkali-activated materials, the GWP100a category provides an important starting point for interpreting the contribution results. Sodium metasilicate pentahydrate was responsible for approximately 41.1% of the total GWP100a, followed by MgO with about 23.8% and slag with approximately 14.5%. Although fly ash, red mud and ceramic powder were incorporated into the mixture, their contributions to GWP remained comparatively low under the adopted modelling assumptions. Since these materials were treated through a cut-off approach, the impacts assigned to them were limited mainly to processes such as drying, grinding or transport where applicable, rather than to the full upstream production burden. As a result, the carbon footprint of the ambient-cured reference mixture was shaped primarily by the solid activator and MgO, rather than by the waste-derived components.

The resource-related categories showed a similar dependence on the activator system. In abiotic depletion, which reflects the consumption of non-living mineral and elemental resources, sodium metasilicate pentahydrate accounted for approximately 69.5% of the total impact. Sodium carbonate represented the second largest share at about 12.6%, followed by slag with approximately 7.9%. The limited contribution of the waste-derived inputs shows that mineral resource depletion was driven mainly by the solid activators rather than by the precursor materials. A slightly more distributed pattern was observed for abiotic depletion of fossil fuels. In this category, sodium metasilicate pentahydrate contributed approximately 47.0% of the total impact, while slag and MgO accounted for about 15.8% and 13.0%, respectively. The higher shares of slag and MgO in fossil fuel depletion suggest that, beyond the activator itself, energy-intensive mineral inputs also played a meaningful role in shaping the energy demand of the reference matrix.

The dominance of sodium metasilicate pentahydrate became even more pronounced in ozone layer depletion, where it accounted for approximately 86.9% of the total impact. The remaining materials contributed only marginally in comparison. Such a concentrated contribution suggests that this category was strongly influenced by the background dataset selected for sodium metasilicate pentahydrate and its associated upstream production processes. Therefore, the ozone depletion result should be interpreted not only as a mixture-design outcome, but also as a reflection of the environmental profile embedded in the selected activator dataset.

Toxicity-related categories also highlighted the importance of the activator system, although the contribution pattern varied across human, freshwater, marine and terrestrial toxicity. In human toxicity, sodium metasilicate pentahydrate contributed approximately 57.1% of the total impact, followed by MgO at around 16.8% and slag at about 8.7%. Sodium carbonate also made a noticeable contribution. This distribution shows that the toxicity burden was mainly associated with the production of the solid activator and mineral additives, rather than with the waste-derived materials.

For aquatic ecotoxicity, MgO became more prominent. In freshwater aquatic ecotoxicity, sodium metasilicate pentahydrate and MgO accounted for approximately 45.9% and 31.3% of the total impact, respectively. The relatively high contribution of MgO distinguishes this category from those dominated almost exclusively by the activator system. Marine aquatic ecotoxicity followed a related pattern, although sodium metasilicate pentahydrate again represented the largest share, accounting for approximately 52.1% of the total impact. MgO contributed around 21.1%, while slag accounted for about 10.1%. These results show that ecotoxicity-related burdens were concentrated mainly in sodium metasilicate pentahydrate and MgO, with slag acting as a secondary contributor.

In terrestrial ecotoxicity, the contribution profile shifted back toward a stronger activator dominance. Sodium metasilicate pentahydrate accounted for approximately 67.4% of the total impact, followed by sodium carbonate at about 10.3% and MgO at around 7.3%. Transport and mixing electricity remained comparatively small contributors, suggesting that the terrestrial toxicity profile was primarily shaped by material production rather than by the processing energy used at the laboratory scale.

The emission-related categories showed a more distributed contribution structure. In photochemical oxidation, which is associated with the formation potential of photochemical oxidants, sodium metasilicate pentahydrate remained the largest contributor with approximately 36.8%. However, MgO and slag also had substantial shares, contributing about 18.7% and 17.8%, respectively. Unlike ozone depletion or abiotic depletion, this category was therefore not controlled by a single input alone, but by the combined contribution of the activator, MgO and slag. A comparable distribution was observed for acidification, where sodium metasilicate pentahydrate contributed approximately 40.7%, while slag and sodium carbonate accounted for about 18.7% and 13.1%, respectively. The contribution of slag in this category suggests that acidification-related emissions were influenced not only by the activator system, but also by the production and supply of the slag component.

Eutrophication followed the broader hotspot pattern observed across the study. Sodium metasilicate pentahydrate contributed approximately 50.2% of the total impact, while MgO and slag represented approximately 15.9% and 11.8%, respectively. The contribution of these three inputs shows that nutrient enrichment-related impacts were again mainly associated with the activator system and mineral additives, rather than with the cut-off waste-derived materials.

Taken together, the contribution analysis shows that the environmental performance of the ambient-cured reference mortar was primarily governed by sodium metasilicate pentahydrate. Its role was especially important for GWP100a, where it represented the largest single contribution to the carbon footprint of the mixture, as well as for ozone layer depletion, abiotic depletion and several toxicity-related categories. MgO, slag and sodium carbonate also contributed substantially in selected categories, particularly fossil fuel depletion, GWP, aquatic ecotoxicity, photochemical oxidation, acidification and eutrophication. In contrast, fly ash, red mud and ceramic powder had limited environmental burdens under the adopted cut-off modelling approach. From an optimization perspective, the most effective strategy would therefore be to reduce the amount or environmental intensity of the solid activator system, especially sodium metasilicate pentahydrate. Further improvements could also be achieved by optimizing MgO and slag-related inputs, particularly in categories where their contributions become environmentally relevant.

**Figure 25:**
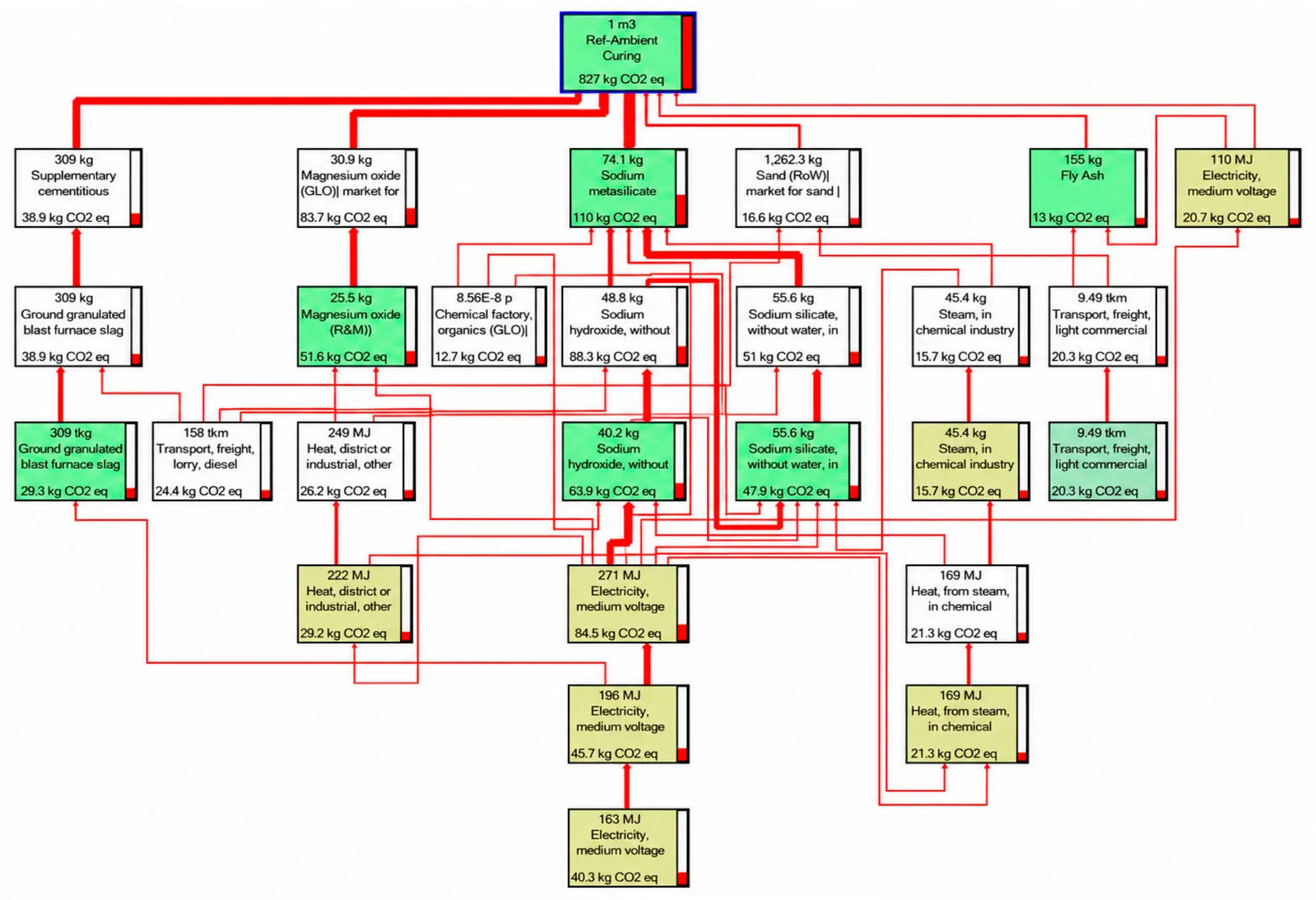
SimaPro process network for 1 m^3^ of ambient-cured reference mortar.

The SimaPro process network of the ambient-cured reference mixture is shown in Figure 25. The diagram visually confirms the contribution analysis, indicating that sodium metasilicate pentahydrate, MgO, slag and sodium carbonate were the main contributors to the environmental profile of 1 m^3^ of reference mortar, while the waste-derived inputs and mixing electricity had comparatively lower contributions.

## 4. Discussion

The experimental results showed that heat curing produced broad and consistent changes in the matrix-related properties of the one-part geopolymer mortars. In comparison to ambient curing, heat curing increased the 28-day compressive strength by 39.5–71.8%, flexural strength by 29.2–134.4%, and UPV by 20.5–36.4%, while reducing sorptivity by 12.2–35.7%. Thermal curing produced a denser and more cohesive matrix than ambient curing, which explains the systematic improvements observed in strength, UPV, abrasion resistance, and capillary transport resistance. However, the influence of fiber type was property-specific. Therefore, heat curing is interpreted as producing the most consistent overall shift across the measured matrix-related indicators, whereas fiber selection controlled the balance among individual performance attributes.

It is important to note that the observed performance differences among the mixtures cannot be attributed solely to the chemical nature of the fibers. The fibers differ significantly in their geometric and mechanical properties (as presented in Table 1), which directly govern their interaction with the geopolymer matrix. For instance, the aspect ratios (length-to-diameter) of basalt (L/D = 600) and PVA (L/D ≈ 205) fibers are substantially higher than that of micro-steel fibers (L/D = 40). In the fresh state, fibers with higher aspect ratios and smaller diameters introduce a significantly larger number of individual fibers per unit volume. This drastically increases the total specific surface area, fundamentally explaining the pronounced reduction in flowability observed in the PVA and basalt mixtures compared to the micro-steel mixtures. In the hardened state, the high aspect ratio and larger contact area of PVA and basalt fibers enhance micro-crack bridging efficiency at the microscopic level [46]. Conversely, micro-steel fibers, despite having a much lower aspect ratio, possess a significantly larger diameter (0.15 mm) and high rigidity. This geometry, combined with their high elastic modulus, provides superior macroscopic dowel action and load-transfer capacity across the interfacial transition zone, which directly accounts for the highest compressive strengths observed in the micro-steel reinforced specimens [47].

Building upon these geometric effects, fiber type introduced a clear performance differentiation. PVA fibers caused the largest reduction in flowability, which may be attributed to their hydrophilic nature and higher effective surface area in the fresh matrix. More generally, fibers with a high specific surface area, such as PVA and basalt fibers, can retain part of the mixing water and binder paste around their surfaces, thereby increasing internal friction within the fresh mortar [48]. As the fiber volume fraction increases, the risk of fiber agglomeration and non-uniform dispersion may also increase, potentially creating localized weak interfacial regions and microvoids [46,48]. Such defects can adversely affect UPV and increase water uptake when fiber dispersion becomes inadequate [46]. In the present study, the lower flowability of 0.8PVA and the decrease in basalt-fiber flowability from 180 to 165 mm as the dosage increased from 0.4% to 0.8% are consistent with this dosage-sensitive fresh-state behavior, although the formation of microvoids was not directly quantified. Despite this penalty in workability, the PVA-reinforced mixtures achieved the most favorable flexural and abrasion-related response, especially at the higher dosage under heat curing. This behavior is consistent with the crack-bridging role of PVA fibers reported in fiber-reinforced geopolymer systems [7,8], and it indicates that tensile crack control and surface stabilization were effectively improved in the present mortar. The superior abrasion performance of 0.8PVA can be explained by a combination of three mechanisms. First, the hydrophilic surface of PVA promotes possible interfacial interaction with the aluminosilicate gel at the ITZ, enabling fibers to carry tensile stress generated by the abrasive wheel rather than debonding prematurely. Second, the crack-bridging action of well-dispersed PVA fibers restricts the lateral propagation of surface microcracks initiated by abrasive contact, limiting the depth of material removal per wear cycle. Third, the near-surface zone of PVA-reinforced mortars benefits from increased fiber density, which provides physical confinement of the surface layer and stabilizes aggregate particles against plucking. Under heat curing, 0.8PVA recorded the lowest thickness loss of 0.63 mm, compared to 1.18 mm for 0.8PVA under ambient curing and 0.73 mm for 0.8MS under heat curing, confirming that the PVA–matrix bond was further strengthened by elevated-temperature treatment. The abrasion wear values observed in the present study for the unfibered reference (0.65 mm under heat curing) are consistent with the range of 0.21–0.87 mm reported by Sor et al. [42] for GGBFS-based geopolymer mortars incorporating ceramic powder under Böhme testing conditions, confirming that the present system operates within a well-established performance envelope for geopolymer flooring applications.

Micro-steel fibers exhibited a different response. These mixtures showed the highest flowability values and the highest compressive strengths, particularly under heat curing. This suggests that micro-steel fibers did not hinder particle rearrangement to the same extent as the polymeric fibers and that they contributed positively to stress transfer under compression. The compressive strength improvement can be attributed to the load-transfer efficiency of micro-steel fibers across the interfacial transition zone (ITZ), whereby the high elastic modulus of steel (200 GPa) enables effective stress redistribution before crack propagation initiates. The high stiffness and load-transfer capacity of steel fibers are also commonly associated with improved flexural toughness, energy absorption and post-cracking ductility in fiber-reinforced matrices [30] although these responses were not directly quantified in the present study. At the fiber–matrix ITZ, the mechanical interlocking between the steel fiber surface and the geopolymer gel phase arrests microcrack opening under compressive loading, thereby delaying catastrophic failure and raising the apparent compressive strength. The 0.8MS mixture reached 64.8 MPa after 7 days of heat curing and 72–73 MPa after 28 days, representing improvements of approximately 4–5% and 2% respectively over the reference under heat curing. These values are consistent with the compressive strength increases of 15–25% reported for steel-fiber-reinforced alkali-activated slag systems by Al-Mashhadani et al. [35] and Arslan et al. [7], with the comparatively moderate gain in the present system attributable to the multi-precursor matrix chemistry and the finer fiber geometry employed. However, the transport-related and abrasion results demonstrate that the mixture providing the highest compressive strength was not always the one with the lowest water uptake or the best wear resistance. This point is important for practice because one-part geopolymer mortars intended for field use must often satisfy multiple performance requirements simultaneously.

Basalt fiber mixtures showed a more balanced response between fresh-state behavior, strength, and water transport. Under heat curing, basalt-containing mortars exhibited low water absorption and low sorptivity while maintaining adequate mechanical performance. This suggests that basalt fibers were compatible with the developed matrix and that the resulting interfacial condition did not severely compromise matrix compactness. As an inorganic mineral fiber with a SiO_2_- and Al_2_O_3_-rich composition, basalt may also exhibit favorable chemical affinity with an aluminosilicate-based geopolymer matrix, potentially supporting effective interfacial bonding [48]. This chemical similarity may partly contribute to the balanced mechanical and transport response observed for the basalt-reinforced mixtures, although direct characterization of the basalt fiber–matrix interfacial chemistry was not performed in this study.

The precursor balance adopted in the present matrix may also be relevant to its mechanical and durability-related performance. Low-calcium, silica- and alumina-rich residues, including many ceramic and brick powders, can favor the development of a stable aluminosilicate gel network and have been associated with improved durability performance. However, excessive replacement of highly reactive calcium-rich precursors such as GGBFS can dilute the reactive phase assemblage, increase porosity and consequently reduce compressive strength [25]. In the present formulation, ceramic powder and red mud were each incorporated at 10%, while GGBFS remained the dominant precursor at 50%. This balance may have limited excessive dilution of the reactive slag fraction while allowing substantial incorporation of waste-derived materials.

The role of MgO in the present system warrants specific attention. Reactive MgO was incorporated at 5 wt.% as a supplementary component to regulate reaction kinetics and promote microstructural refinement. Since MgO content was kept constant in all mixtures, the present experimental design does not isolate the independent contribution of MgO. Therefore, the discussion of MgO-related hydrotalcite formation and matrix refinement should be understood as a literature-supported interpretation rather than a mechanism directly verified by variable MgO contents in this study. In alkali-activated slag systems, MgO participates in the formation of hydrotalcite-like phases (Mg–Al layered double hydroxides), which act as secondary binding phases that fill interstitial pore space and contribute to matrix densification [11,12]. The formation of these phases is particularly pronounced at elevated temperatures, which may help explain why the performance advantage associated with heat curing was more consistent across all mixtures than might be expected from curing temperature alone. Additionally, MgO reacts with the calcium-rich slag phase to promote supplementary C–S–H formation, reducing effective capillary porosity and improving the continuity of the geopolymer binder network [13]. The UPV results support this interpretation: heat-cured specimens consistently exhibited UPV values above 3500 m/s, indicating a compact and well-connected internal structure that reflects the combined contribution of possible MgO-related matrix refinement and elevated-temperature reaction acceleration. The slightly higher sorptivity of micro-steel fiber mixtures relative to PVA and basalt mixtures under heat curing may reflect a disruption of the MgO-promoted densification near fiber surfaces, where the relatively high stiffness of steel creates stress concentrations at the ITZ that partially offset the pore-filling effect of the MgO-derived phases [11,12,13]. In addition, exposure to elevated temperatures (500–750 °C) promotes the formation of highly thermally stable akermanite phases in MgO-containing systems, which has been reported to improve the material’s resistance to high-temperature conditions [30].

The microstructural characterization supports the mechanical and transport results. XRD showed that all heat-cured mixtures retained a broadly similar mineralogical framework, indicating that fiber incorporation did not produce a fundamentally different crystalline reaction path. FTIR confirmed the formation of an aluminosilicate reaction network in all heat-cured mixtures, while SEM/EDS revealed a compact but heterogeneous matrix containing reacted regions together with residual solid features. To provide a more meaningful interpretation of the reaction products, quantitative elemental ratios were evaluated from the EDS atomic percentages. In the analyzed regions of the selected specimens, the Si/Al ratios varied between approximately 2.60 and 2.93. The Si/Al ratios are consistent with the formation of a dense, highly polymerized aluminosilicate network, which is commonly associated with mature N-A-S-H-type geopolymeric gels; however, the literature also indicates that values above this range do not necessarily maximize strength and may reflect excess silica in some systems [49,50,51,52]. The Ca/Si ratios of 0.41–0.88 suggest the presence of a calcium-rich hybrid gel network, likely involving coexisting C-(A)-S-H/C-(N)-A-S-H and N-A-S-H-type products, as commonly reported in slag-rich alkali-activated systems [43,53].

Taken together, these findings indicate that the observed performance differences were governed primarily by matrix densification, pore structure refinement, and fiber–matrix interaction rather than by the formation of entirely new crystalline products [43,44].

According to the characterized LCA results, thermal curing at 80 °C for 24 h increased the Global Warming Potential (GWP100a) of the reference mixture from 267.37 kg CO_2_-eq to 287.67 kg CO_2_-eq, corresponding to a 7.60% increase. Although this suggests that oven curing introduces an additional environmental burden, the relatively limited magnitude of the increase suggests that the carbon footprint of the system is not primarily controlled by curing electricity. Instead, the dominant contribution is associated with the raw materials, particularly the alkaline activator system. This interpretation is consistent with previous studies on geopolymer and alkali-activated materials, which have repeatedly identified alkaline activators as one of the main environmental hotspots [18,54]. Sodium silicate, in particular, is known to have a high embodied carbon due to the energy-intensive manufacturing process, which involves high-temperature production stages. Commercial sodium silicate has been reported to generate approximately 1860 kg CO_2_ per ton [40], and in some geopolymer binder systems it may account for more than half of the total carbon emissions. Similarly, the combined contribution of sodium silicate and sodium hydroxide activators can approach nearly 200 kg CO_2_-eq within the total GWP of the mixture [38,39]. This finding is also consistent with the broader LCA literature, in which sodium silicate-based activators have repeatedly been identified as the most carbon-intensive components of geopolymer production, accounting for approximately 50–70% of embodied energy (EE) and GWP in some systems [26]. This highlights the activator system as an important target for further environmental optimization. From a circular-economy perspective, future studies could therefore investigate alternative activators with lower embodied impacts, including sodium silicate synthesized from waste glass, as a partial or complete substitute for commercially produced sodium silicate. Such an approach may further reduce both the carbon footprint and material cost of one-part geopolymer systems while simultaneously promoting the valorization of silica-rich waste streams [26].

Despite the increase in GWP to 287.67 kg CO_2_-eq and fossil fuel consumption to 2953.52 MJ following thermal curing, the obtained environmental impact values still remain highly sustainable when compared with conventional OPC The production of traditional OPC alone is responsible for approximately 7–10% of global greenhouse gas emissions, with an estimated release of nearly 850 kg CO_2_ per ton of cement produced [20,37]. Detailed LCA studies reported in the literature have shown that the GWP of 1 m^3^ of OPC-based concrete reaches approximately 597.54 kg CO_2_-eq, whereas geopolymer systems can exhibit values as low as 133–148 kg CO_2_-eq [55,56]. From a broader perspective, geopolymer binders have been demonstrated to reduce carbon emissions by approximately 60–80% and significantly decrease overall energy consumption compared to OPC-based systems [19,22]. The measured value of 287.67 kg CO_2_-eq in the present study numerically indicates that, despite the application of an energy-intensive oven-curing process, geopolymers still constitute a highly environmentally friendly and low-carbon alternative to conventional cementitious materials.

Sodium carbonate has emerged in the literature as one of the most environmentally friendly (“greener”) and sustainable solid activator alternatives for geopolymer systems [15,17]. The carbon emissions associated with sodium carbonate production are reported to range between approximately 250 and 300 kg CO_2_ per ton, which is considerably lower than those of conventional alkaline activators. In addition, its embodied energy is remarkably low, at only about 1.35 MJ/kg [22]. Sodium carbonate (Na_2_CO_3_) is generally produced through the Solvay (ammonia-soda) process, which is regarded as highly carbon-efficient due to its capability to capture and reutilize CO_2_ from industrial exhaust or flue gases during the production cycle [17].

According to LCA analyses reported in the literature [15], the carbon footprint of conventional anhydrous sodium silicate (Na_2_SiO_3_-anhydrous) production is exceptionally high, reaching approximately 1860 kg CO_2_ per ton. In contrast, the carbon emissions associated with sodium metasilicate pentahydrate (Na_2_SiO_3_·5H_2_O) decrease to around 1530 kg CO_2_ per ton. Moreover, for hydrated forms with higher water content, such as the nonahydrate version, this value can be reduced further to nearly 870 kg CO_2_ per ton [15]. In addition, the highly viscous and corrosive liquid activators commonly used in conventional two-part geopolymer systems, namely liquid sodium silicate and NaOH, not only generate additional transportation-related emissions and safety concerns during storage and handling, but also complicate the control of the water-to-binder ratio within the mixture [14,16].

According to the LCA results obtained in the present study, the GWP associated with the solid activators used in the reference mixture cured at ambient temperature was calculated as 109.97 kg CO_2_-eq for sodium metasilicate pentahydrate and 10.69 kg CO_2_-eq for sodium carbonate. The combined environmental burden originating from these chemicals, corresponding to approximately 120.66 kg CO_2_-eq, accounts for nearly 45% of the total carbon footprint of the reference mixture, which was determined to be 267.37 kg CO_2_-eq. Previous studies [56,57] in the literature have reported that alkaline activators contribute between 55% and 74% of the total carbon emissions associated with geopolymer binder production. In another recent study [22], activators were shown to be responsible for nearly two-thirds (66%) of the total emissions, while sodium silicate alone accounted for approximately 50% of the overall environmental burden. Therefore, the comparatively lower activator-related environmental impact observed in the present study (45%) suggests that the hybrid activator design based on sodium metasilicate pentahydrate and sodium carbonate provides a successful optimization strategy in terms of carbon efficiency. It should be noted that this environmental evaluation was explicitly conducted as a screening life-cycle assessment. Consequently, the absolute values are inevitably influenced by the adopted assumptions regarding transportation distances (e.g., 30 km for local by-products), grid electricity mixes, and the selection of proxy datasets. While a comprehensive sensitivity analysis falls outside the scope of this baseline assessment, the substantial margin by which the solid activators dominate the environmental footprint—accounting for over 41% of the total GWP—suggests that realistic variations in local transport distances or mixing electricity would not fundamentally alter the primary hotspot identification. Nevertheless, future scale-up studies should incorporate detailed sensitivity and uncertainty analyses to capture site-specific supply chain variations.

The main scientific and practical contribution of this study lies in demonstrating that a one-part geopolymer mortar incorporating slag, fly ash, ceramic powder, red mud, and MgO can be tailored toward different application targets through controlled fiber selection and curing. Although curing at 80 °C for 24 h significantly improves performance, this regime is more practical for precast or factory-controlled production than for large-scale in situ construction, where ambient-cured one-part systems are being actively investigated as a lower-energy alternative [25,58]. The combined use of industrial residues, dry activators, and reinforcement-specific performance optimization offers a realistic pathway for producing more sustainable mortar systems suited to repair, precast, and wear-exposed applications. Future work should also quantify initial and final setting times and open-time behavior, particularly considering the combined influence of the slag-rich precursor system, reactive MgO, and solid alkaline activators, together with shrinkage, freeze–thaw resistance, sulfate resistance, and longer-term microstructural development under ambient curing.

## 5. Conclusions

Based on the experimental findings, the following conclusions can be drawn:

(1) Heat curing at 80 °C for 24 h enhanced the mechanical performance by increasing the 28-day compressive strength by 39.5–71.8%, flexural strength by 29.2–134.4%, and UPV by 20.5–36.4% relative to ambient curing. Sorptivity and Böhme thickness loss decreased for all mixtures, but 24 h water absorption improved only for PVA and basalt mixtures.
(2) Fiber type and dosage produced property-specific responses and had a clear influence on both fresh and hardened properties with the effects of curing regime. For instance, PVA fibers caused the largest reduction in flowability, with the 0.8PVA mixture showing a 23.6% lower flow diameter than the reference. In contrast, micro-steel fibers increased flowability, indicating that fresh-state behavior depended strongly on fiber surface characteristics, geometry, and dispersion.
(3) The highest compressive strength was obtained in 0.8MS reaching 64.8 MPa after 7 days of heat curing. At 28 days, the reference and 0.4MS mixtures both reached 73 MPa, while 0.8MS reached 72 MPa. The highest heat-cured flexural strength, 7.5 MPa, was recorded for both 0.8PVA and 0.4MS.
(4) Under heat curing, PVA- and basalt-fiber mixtures generally exhibited the most favorable water-transport response, while 0.8PVA recorded the lowest Böhme thickness loss of 0.63 mm. Thus, the mixtures giving the highest compressive strengths were not necessarily those providing the best transport and wear performance.
(5) XRD, FTIR, and SEM/EDS analyses suggested the formation of a reacted aluminosilicate matrix with residual crystalline features and a compact heterogeneous microstructure under heat-cured conditions.
(6) A cradle-to-gate screening LCA conducted for the non-fiber-reinforced reference formulation showed that heat curing increased the GWP by only 7.60%, from 267.37 to 287.67 kg CO_2_-eq. The environmental profile was governed mainly by raw materials, particularly the solid activator system, which accounted for approximately 45% of the total carbon footprint. Within this limited LCA scope, these results indicate the potential of the developed one-part geopolymer matrix to reduce clinker dependency and support lower-carbon binder development, although broader environmental comparisons including fiber-reinforced mixtures require further assessment.

Overall, the results demonstrate that curing conditions and fiber selection can be used to adjust the mechanical, physical and durability-related performance of the investigated one-part geopolymer mortar. Nevertheless, service-related properties, including shrinkage, cracking, bond strength, and freeze–thaw resistance, should be evaluated before specific practical applications are proposed.

## Figures and Tables

**Table 1:** Physical and mechanical properties of the fibers used in this study.

Fiber Type	Length/Diameter (mm/mm)	Tensile Strength (MPa)	Density (g/cm^3^)
Micro-steel	6/0.15	2100	7.85
PVA	8/0.039	1620	1.30
Basalt	12/0.020	4100	2.80

**Table 2:** Chemical composition of the raw precursor materials (wt.%). Source: XRF analysis reports (present study); MgO from supplier datasheet [24].

Oxide (wt.%)	GGBFS	Fly Ash	Ceramic	Red Mud	MgO
SiO_2_	35.92	45.11	60.42	11.04	3.11
Al_2_O_3_	9.03	22.30	16.01	12.01	0.15
CaO	53.06	6.69	15.38	24.23	1.71
Fe_2_O_3_	1.99	18.82	8.20	45.44	0.35
MgO	—	0.21	—	—	84.55
TiO_2_	—	2.02	—	5.85	—
SO_3_	—	1.89	—	0.20	—
K_2_O	—	1.30	—	0.30	—
LOI	—	—	—	—	10.13

**Table 3:** Mix proportions of the geopolymer mortars used for performance testing.

Mix ID	Slag (%)	Fly Ash (%)	Ceramic (%)	Red Mud (%)	MgO (%)	Fiber (%)	Na_2_SiO_3_·5H_2_O (%)	Na_2_CO_3_ (%)	W/B	Sand/B
Ref.	50	25	10	10	5	0	12	4	0.32	2:1
0.4PVA	50	25	10	10	5	0.4	12	4	0.32	2:1
0.8PVA	50	25	10	10	5	0.8	12	4	0.32	2:1
0.4BF	50	25	10	10	5	0.4	12	4	0.32	2:1
0.8BF	50	25	10	10	5	0.8	12	4	0.32	2:1
0.4MS	50	25	10	10	5	0.4	12	4	0.32	2:1
0.8MS	50	25	10	10	5	0.8	12	4	0.32	2:1

**Table 4:** Impact assessment categories and interpretation approach used in the LCA.

Item	Assumption Adopted in This Study
Impact assessment method and categories	Environmental impacts were calculated using the CML-IA baseline method. The impact categories included abiotic depletion, abiotic depletion of fossil fuels, global warming potential (GWP100a), ozone layer depletion, human toxicity, freshwater aquatic ecotoxicity, marine aquatic ecotoxicity, terrestrial ecotoxicity, photochemical oxidation, acidification and eutrophication.
Interpretation of ambient scenario	For the ambient-cured reference mixture, the material and process contribution to each impact category was calculated and presented in tabular form.
Comparison of heat curing scenario	For the heat-cured reference mixture, the total characterized results for each impact category were compared with the ambient-cured reference mixture, and the percentage variation was graphically evaluated.

**Table 5:** The main assumptions adopted for process modelling, transportation and dataset selection.

Item	Assumption Adopted in This Study
Laboratory mixing machine	Laboratory-scale bench-top mixer was used. Mixing electricity was included in the LCA.
Heat curing	For heat-cured specimens, oven curing at 80 °C for 24 h was considered as an additional electricity input.
Packaging	Packaging was excluded because its effect was assumed to be negligible.
Cementitious precursor: GGBFS	Modelled using the corresponding Ecoinvent 3.11 market dataset.
Fly ash	Modelled as a cut-off by-product. Transport: 30 km by light commercial vehicle.
Red mud	Modelled as a cut-off by-product. Drying and grinding electricity and Transport included as a project-specific assumption.
Ceramic powder	Modelled as a cut-off by-product. Drying electricity and Transport: 30 km by light commercial vehicle.
MgO	Modelled using the Ecoinvent 3.11 magnesium oxide dataset.
Sodium metasilicate pentahydrate	Modelled using the Ecoinvent 3.11 market dataset for sodium metasilicate pentahydrate powder.
Sodium carbonate	Modelled using the Ecoinvent 3.11 market dataset for soda ash, light.
Fine aggregate	Modelled using the Ecoinvent 3.11 market dataset for sand/silica sand.
Water	Modelled using the Ecoinvent 3.11 market dataset for tap water.

**Table 6:** Flowability of fresh geopolymer mortar mixtures.

Mix ID	Flowability (mm)
Ref	174 ± 5
0.4PVA	152 ± 2
0.8PVA	133 ± 6
0.4BF	180 ± 7
0.8BF	165 ± 5
0.4MS	190 ± 8
0.8MS	201 ± 5

**Table 7:** Flexural strength of geopolymer mortars after 7 days under ambient curing and heat curing (80 °C).

Mix ID	Flexural—Ambient (MPa)	Flexural—Heat (MPa)
Ref	1.9 ± 0.1	4.4 ± 0.2
0.4PVA	3.2 ± 0.1	5.0 ± 0.1
0.8PVA	3.5 ± 0.2	5.0 ± 0.1
0.4BF	2.4 ± 0.0	4.2 ± 0.3
0.8BF	2.8 ± 0.1	6.2 ± 0.3
0.4MS	2.4 ± 0.1	5.3 ± 0.3
0.8MS	2.0 ± 0.1	4.8 ± 0.2

**Table 8:** Flexural strength and UPV of geopolymer mortars after 28 days under ambient curing and heat curing (80 °C).

Mix ID	Flexural—Ambient (MPa)	UPV—Amb. (m/s)	Flexural—Heat (MPa)	UPV—Heat (m/s)
Ref	2.7 ± 0.1	2807.0 ± 20.0	5.6 ± 0.1	3636.4 ± 15.4
0.4PVA	4.8 ± 0.2	2711.9 ± 9.9	6.2 ± 0.3	3595.5 ± 12.3
0.8PVA	5.5 ± 0.1	2758.6 ± 24.9	7.5 ± 0.3	3653.0 ± 15.1
0.4BF	3.0 ± 0.1	2666.7 ± 10.1	6.7 ± 0.1	3636.4 ± 11.9
0.8BF	3.7 ± 0.1	2882.9 ± 12.2	6.4 ± 0.2	3524.2 ± 17.8
0.4MS	3.2 ± 0.1	2990.7 ± 15.4	7.5 ± 0.2	3603.6 ± 15.4
0.8MS	3.4 ± 0.3	2735.0 ± 19.9	7.0 ± 0.3	3516.5 ± 18.0

**Table 9:** Compressive strength of geopolymer mortars after 7 days under ambient curing and heat curing (80 °C).

Mix ID	Comp.—Ambient (MPa)	Comp.—Heat (MPa)
Ref	33.0 ± 0.5	62.2 ± 0.3
0.4PVA	34.9 ± 0.4	52.5 ± 0.2
0.8PVA	28.8 ± 0.3	46.0 ± 1.0
0.4BF	30.8 ± 0.8	49.0 ± 1.2
0.8BF	32.1 ± 0.2	48.0 ± 0.8
0.4MS	39.5 ± 0.9	56.8 ± 1.0
0.8MS	40.8 ± 0.2	64.8 ± 1.0

**Table 10:** Compressive strength of geopolymer mortars after 28 days under ambient curing and heat curing (80 °C).

Mix ID	Comp.—Ambient (MPa)	Comp.—Heat (MPa)
Ref	42.5 ± 0.5	73.0 ± 3.0
0.4PVA	38.0 ± 1.2	53.0 ± 1.0
0.8PVA	37.5 ± 0.6	54.0 ± 1.3
0.4BF	35.0 ± 0.7	59.0 ± 1.5
0.8BF	37.5 ± 0.6	55.0 ± 0.4
0.4MS	46.7 ± 1.3	73.0 ± 1.0
0.8MS	44.0 ± 1.8	72.0 ± 1.5

**Table 11:** Splitting tensile strength of geopolymer mortars under ambient curing and heat curing (80 °C).

Mix ID	Ambient Curing (MPa)	Heat Curing 80 °C (MPa)
Ref	2.3 ± 0.2	4.3 ± 0.3
0.4PVA	3.5 ± 0.2	4.4 ± 0.1
0.8PVA	3.5 ± 0.1	4.4 ± 0.1
0.4BF	3.6 ± 0.2	4.5 ± 0.2
0.8BF	3.4 ± 0.3	4.1 ± 0.0
0.4MS	3.5 ± 0.1	4.2 ± 0.3
0.8MS	3.4 ± 0.1	4.4 ± 0.3

**Table 12:** Böhme abrasion results of ambient-cured geopolymer mortar mixtures.

Mix ID	Mass Loss (g)	Mass Loss (%)	Thickness Loss (mm)	Thickness Loss (%)
Ref	13.75 ± 0.39	1.84 ± 0.05	1.31 ± 0.04	1.85 ± 0.06
0.4PVA	13.0 ± 0.51	1.81 ± 0.07	1.55 ± 0.04	2.18 ± 0.06
0.8PVA	10.25 ± 0.14	1.45 ± 0.02	1.18 ± 0.02	1.65 ± 0.03
0.4BF	8.5 ± 0.11	1.18 ± 0.02	1.38 ± 0.02	1.94 ± 0.03
0.8BF	9.75 ± 0.12	1.37 ± 0.02	1.54 ± 0.02	2.17 ± 0.03
0.4MS	18.0 ± 0.21	2.46 ± 0.03	1.75 ± 0.02	2.46 ± 0.03
0.8MS	28.5 ± 0.68	3.88 ± 0.09	1.56 ± 0.05	2.20 ± 0.07

**Table 13:** Böhme abrasion results of heat-cured geopolymer mortar mixtures.

**Mix ID**	**Mass Loss (g)**	**Mass Loss (%)**	**Thickness Loss (mm)**	**Thickness Loss (%)**
Ref	12.0 ± 0.49	1.57 ± 0.06	0.65 ± 0.02	0.92 ± 0.03
0.4PVA	11.5 ± 0.22	1.57 ± 0.03	1.33 ± 0.03	1.87 ± 0.04
0.8PVA	7.5 ± 0.33	1.02 ± 0.04	0.63 ± 0.02	0.88 ± 0.03
0.4BF	7.5 ± 0.20	1.03 ± 0.03	1.18 ± 0.05	1.65 ± 0.07
0.8BF	8.5 ± 0.13	1.18 ± 0.02	1.05 ± 0.07	1.48 ± 0.10
0.4MS	7.75 ± 0.10	1.05 ± 0.01	0.94 ± 0.03	1.32 ± 0.04
0.8MS	13.0 ± 0.24	1.75 ± 0.03	0.73 ± 0.01	1.02 ± 0.01

**Table 14:** Water absorption after 24 h of geopolymer mortars under ambient curing and heat curing (80 °C).

Mix ID	WA_24h_ (%)—Ambient Curing	WA_24h_ (%)—Heat Curing (80 °C)
Ref	4.18 ± 0.08	4.40 ± 0.05
0.4PVA	4.16 ± 0.08	2.19 ± 0.06
0.8PVA	4.19 ± 0.05	2.67 ± 0.10
0.4BF	4.17 ± 0.12	2.12 ± 0.03
0.8BF	4.46 ± 0.05	2.59 ± 0.06
0.4MS	4.28 ± 0.03	4.43 ± 0.08
0.8MS	4.32 ± 0.05	4.42 ± 0.02

**Table 15:** Sorptivity of geopolymer mortars under ambient curing and heat curing (80 °C).

Mix ID	Sorptivity—Ambient (mm/min^0.5^)	Sorptivity—Heat (mm/min^0.5^)	R^2^—Ambient	R^2^—Heat
Ref	0.178 ± 0.004	0.147 ± 0.004	0.977	0.999
0.4PVA	0.171 ± 0.002	0.110 ± 0.003	0.961	0.994
0.8PVA	0.171 ± 0.001	0.122 ± 0.001	0.964	0.992
0.4BF	0.173 ± 0.001	0.112 ± 0.002	0.975	0.997
0.8BF	0.174 ± 0.001	0.118 ± 0.002	0.958	0.997
0.4MS	0.177 ± 0.001	0.150 ± 0.003	0.968	0.999
0.8MS	0.172 ± 0.003	0.151 ± 0.002	0.950	1.000

**Table 16:** Main FTIR absorption bands identified for the heat-cured geopolymer mortars.

Specimen ID	Mix ID	Main Absorption Bands (cm^−1^)	Main Interpretation
M1	Ref	975.898	Main Si–O–T asymmetric stretching band of geopolymeric gel
M2	0.8PVA	1416, 993, 778	Carbonate-related band; main Si–O–T band; possible Si–O–Si/residual silica-related vibration
M3	0.8BF	1420, 965	Carbonate-related band; main Si–O–T band of geopolymeric framework
M4	0.8MS	971	Main Si–O–T asymmetric stretching band of geopolymeric gel

**Table 17:** Characterized environmental impacts of the ambient-cured and heat-cured reference mixtures.

Impact Category	Unit	Ref-Ambient Curing	Ref-Heat Curing	Ratio (%)
		Total	Total	
Abiotic depletion	kg Sb eq	0.00239	0.00241	0.84
Abiotic depletion (fossil fuels)	MJ	2726.44	2953.53	8.33
Global warming (GWP100a)	kg CO_2_ eq	267.37	287.68	7.60
Ozone layer depletion (ODP)	kg CFC-11 eq	8.69 × 10^−6^	8.80 × 10^−6^	1.25
Human toxicity	kg 1,4-DB eq	324.29	334.47	3.14
Fresh water aquatic ecotox.	kg 1,4-DB eq	238.88	249.63	4.50
Marine aquatic ecotoxicity	kg 1,4-DB eq	395,923.96	429,201.07	8.40
Terrestrial ecotoxicity	kg 1,4-DB eq	2.154	2.210	2.58
Photochemical oxidation	kg C_2_H_4_ eq	0.0591	0.0623	5.41
Acidification	kg SO_2_ eq	1.199	1.283	7.00
Eutrophication	kg PO4^3−^ eq	0.456	0.503	10.47

**Table 18:** Contribution of individual materials and processes to the characterized environmental impacts of the ambient-cured reference mortar.

Impact Category	Unit	Total	Slag	Ceramic Powder	Red Mud	MgO	Sodium Metasilicate Pentahydrate	Water	Sand	Sodium Carbonate	Fly Ash	Electricity, Medium Voltage
Abiotic depletion	kg Sb eq	0.00239	0.000188	3.24 × 10^−5^	3.17 × 10^−5^	2.53 × 10^−5^	0.00166	1.08 × 10^−6^	6.47 × 10^−5^	0.000300	8.09 × 10^−5^	3.99 × 10^−6^
Abiotic depletion (fossil fuels)	MJ	2726.43	429.35	66.94	70.65	354.11	1280.10	2.62	207.86	102.65	166.75	45.42
Global warming (GWP100a)	kg CO_2_ eq	267.37	38.90	5.23	5.59	63.67	109.97	0.24	16.03	10.69	13.01	4.06
Ozone layer depletion (ODP)	kg CFC-11 eq	8.69 × 10^−6^	3.26 × 10^−7^	5.31 × 10^−8^	5.61 × 10^−8^	2.65 × 10^−7^	7.56 × 10^−6^	5.57 × 10^−8^	1.66 × 10^−7^	6.22 × 10^−8^	1.33 × 10^−7^	2.17 × 10^−8^
Human toxicity	kg 1,4-DB eq	324.29	28.17	4.17	4.30	54.32	185.28	0.18	9.22	26.19	10.41	2.04
Fresh water aquatic ecotox.	kg 1,4-DB eq	238.88	18.30	2.96	3.15	74.80	109.60	0.16	6.84	13.53	7.38	2.15
Marine aquatic ecotoxicity	kg 1,4-DB eq	395,923.96	40,143.29	5332.80	6046.79	83701.95	206,085.69	388.19	11,768.30	22,555.21	13,246.32	6655.42
Terrestrial ecotoxicity	kg 1,4-DB eq	2.154	0.149	0.0218	0.0225	0.158	1.451	0.002	0.063	0.222	0.054	0.011
Photochemical oxidation	kg C_2_H_4_ eq	0.0591	0.0105	0.00162	0.00165	0.0111	0.0218	4.98 × 10^−5^	0.00411	0.00360	0.00405	0.000639
Acidification	kg SO_2_ eq	1.1999	0.235	0.0196	0.0212	0.156	0.511	0.001	0.0818	0.106	0.0488	0.0168
Eutrophication	kg PO4--- eq	0.456	0.0537	0.00772	0.00876	0.07267	0.229	0.00049	0.0237	0.0312	0.0192	0.00954

## Data Availability

The data presented in this study are available from the corresponding author upon reasonable request.
